# Impact of Natural Fermentation on Mineral Composition, Resistant and Non-Resistant Starches, Microbial Diversity, and Global Metabolite Profiles in Commercial Poi from Hawai‘i

**DOI:** 10.3390/metabo15110748

**Published:** 2025-11-18

**Authors:** Nyan Stillwell, Vedbar S. Khadka, Pratibha V. Nerurkar

**Affiliations:** 1 Laboratory of Metabolic Disorders and Alternative Medicine, Department of Molecular Biosciences and Bioengineering (MBBE), College of Tropical Agriculture and Human Resilience (CTAHR), University of Hawai‘i at Manoa (UHM), Honolulu, HI 96822, USA; 2 Bioinformatics Core, Department of Quantitative Health Sciences, John A. Burns School of Medicine (JABSOM), University of Hawai‘i at Manoa (UHM), Honolulu, HI 96813, USA

**Keywords:** poi, taro, fermenting bacteria, resistant starch, lactic acid bacteria, metabolomics

## Abstract

Background/Objectives: Taro (*Colocasia esculenta* L.) is a nutritionally rich and historically significant crop widely consumed in tropical and subtropical regions. Poi, a traditional Hawaiian food made from mashed cooked taro corms, is lauded for its digestibility, prebiotic properties, and potential health benefits. The goal of our study was to evaluate the effects of natural fermentation on the nutritional and metabolic profiles of five commercial poi brands from Hawai‘i. Methods: All poi were fermented at 25 °C for 24 h and 48 h. Resistant starch (RS) and non-resistant starch (NRS) were analyzed using Megazyme assay kits, minerals were analyzed by the EPA 3050B method, fermenting bacteria were analyzed by 16S sequencing, and global metabolites were analyzed using a gas chromatography time-of-flight mass spectrometer. Results: RS and NRS significantly increased in fermented poi, while mineral composition remained unaffected. Untargeted global metabolomic analysis revealed fermentation-induced shifts in metabolite profiles, with increased amino acid concentrations but no changes in essential fatty acids, vitamin E, or flavanols. Fermentation increased the dominance of health-promoting lactic acid bacteria (LAB) such as *Leuconostoc*, *Lactococcus*, *Weissella*, and *Lactobacillus*, known for their health-promoting properties. No significant correlations were identified among the fermenting bacteria and metabolites. This is probably one of the first comprehensive evaluations to identify the impact of fermentation on the starch, mineral, fermenting microbes, and metabolite content of commercial poi and show the presence of 18 amino acids, including nine essential amino acids. Conclusions: Our findings highlight the nutritional and microbiological significance of fermented poi and its potential as a functional food. Further studies are warranted to explore the health benefits and probiotic effects of poi.

## 1. Introduction

Taro (*Colocasia esculenta* L.) belongs to the Araceae family and is one of the oldest cultivated crops in the world, having been grown for more than 9000 years [1,2,3]. Among the different varieties or cultivars of taro, there are two morphological groups: the “eddoe” type (*Colocasia esculenta* var. *antiquorum* syn. *Colocasia esculanta* var. *globulifera*) and the “dasheen” type (*Colocasia esculenta* var. *esculenta*) [4,5]. Many taro cultivars are considered to be intermediates between these two morphological groups [4].

Taro is widely cultivated throughout many parts of the world, particularly in the tropical and subtropical regions, including the Pacific Islands, Asia, the Americas, Africa, and also in the southern Mediterranean region of Turkey [1,6,7]. The worldwide demand for taro is on the rise, and by 2020, global production had reached almost 12.84 million metric tons [8,9]. In the United States, Hawai‘i was the only state growing taro, which has a current production of about 4.8 million pounds and a market value of USD 6.4 million [10]. Historically, more than 150 landraces of preserved taro lineages were grown in Hawai‘i [11]. Generally, taro leaves, corms, and petioles are consumed as food. Although nutritional composition varies based on growth conditions and taro variety and maturity, taro leaves are a rich source of carbohydrates, fat, and fiber and contain high levels of vitamin C, vitamin A, vitamin B, folate, niacin, iron, calcium, gallic acid, chlorogenic acid, caffeic acid, catechins, and antioxidants such as flavonoids, phenolic acids, and proanthocyanidins [12,13].

The consumption of raw taro root or leaves is toxic and can cause burning, pain, and swelling of the mouth or throat, as well as itching and irritation upon contact with bare skin [13]. Raw taro roots and leaves contain natural antinutritional factors such as phytates, oxalates, trypsin inhibitors, and lectins [12,13]. Oxalates are prevalent in all parts of the taro plant and are responsible for the associated toxic symptoms. Cooking typically reduces oxalate levels, allowing taro to be consumed safely [12,13]. Taro is also commonly referred to as dasheen, eddoes, malanga, and cocoyam [1]. In Hawai‘i, taro is referred to as “kalo” in the Hawaiian language, and the tuber (corm) is mostly consumed in the form of poi, while leaves are used to wrap meat and are cooked into traditional dishes called “laulau” [7,11]. Poi, a traditional staple of the Hawaiian diet, is made by pounding the steamed or boiled fresh taro corms with a small amount of water into a smooth paste, using a specific poi pestle known as “pōhaku kuʻi ʻai”, carved from basalt, coral, or wood [2,7]. Modern methods of preparing poi utilize industrial processing techniques to produce larger quantities for retail distribution [2]. In Hawai‘i, the consistency of poi can range from sticky and viscous to watery, based on the water content, and is referred to as either “one-finger”, “two-finger”, or “three-finger”, based on how many fingers are needed to scoop up the poi [14,15,16].

Freshly made poi is usually very bland and starchy tasting and may sometimes have a slightly sweet flavor. Poi is also consumed up to 3–4 days after being prepared, during which time the poi undergoes a fermentation process that changes the pH from approx. 6.3 to 4.5 within the first 24 h and imparts a sour flavor [7,17]. The fermentation of poi is achieved by the natural growth of yeasts and lactic acid bacteria (LAB) such as *Lactobacillus* and *Lactococcus lactis* [7,14]. Fermented poi is usually discarded on the fourth or fifth day after the initial preparation due to declining palatability [7].

Earlier scientific studies indicated that poi is easily digested by both infants and adults, improves food allergy symptoms, food intolerances, and failure-to-thrive in infants [7,18,19], while recent studies suggest that poi has anti-cancer and anti-diabetic properties [2,6,7,15,20]. Enzymatic hydrolysis of cooked and mashed taro corms indicates a medium glycemic index of 63.1 ± 2.5, thereby making it a good dietary carbohydrate alternative for diabetic individuals [6]. Based on taro varieties, the nutrient profile of poi consists primarily of carbohydrates along with small amounts of other nutrients, including vitamins, minerals, antioxidants, and other bioactive molecules such as flavonoids, alkaloids, sterols, tannins, and proteins [6,17,21]. Taro also contains lectins, including tarin, that have been shown to exhibit antitumoral and immunomodulatory properties. Poi is also a good source of fiber, including the non-digestible carbohydrate referred to as resistant starch (RS), and is an excellent source of pre- and probiotics, which provide beneficial effects on the gut [14,15,22,23].

Minerals found in plant-based foods often have very low bioavailability due to the formation of complexes with non-digestible material [24,25,26]. Microbial fermentation via LAB, such as *Lactobacillus plantarum* and *Lactobacillus rhamnosus,* can degrade these complexes and make minerals more bioavailable [24,25,26]. No studies have been conducted to identify the effects of fermentation on vitamins, minerals, and secondary metabolites in poi. The goal of our study was to identify the fermenting bacteria and the effects of fermentation on resistant starch (RS), non-resistant starch (NRS), minerals, and global metabolites in locally available commercial poi.

## 2. Materials and Methods

### 2.1. Commercial Poi

Fresh poi was purchased from local supermarkets in Honolulu, Hawai‘i. Five commonly available and locally prepared fresh poi (Aloha brand poi, Hanalei brand poi, Kokua brand poi, Pomai brand poi, and Taro brand poipoi) were obtained at three separate time points over the course of one month from March to April 2022. Poi obtained from Kokua Market was the only poi that did not have a brand name, but it is always sourced from Reppun Farms located in Waiahole Valley, Oahu, HI. Kokua Market poi will therefore be referred to as Kokua brand poi. Upon purchase, all fresh poi was kept on ice and transported to the laboratory for analysis. Each type of poi was immediately weighed and aliquoted into sterile centrifuge tubes for fermentation. Fresh samples of all unfermented poi were weighed and immediately frozen at −20 °C until further analysis.

### 2.2. Poi Fermentation

Lactic acid bacteria present in taro and the environment can facilitate poi fermentation at a room temperature of 20 °C [15,27]. Our preliminary studies indicated that fermentation of poi at 20 °C for 24 h and 48 h had no effect on the concentrations of resistant starch (RS). We therefore explored the effects of poi fermentation at 25 °C, based on the fact that room temperatures in Honolulu vary between 29.4 °C in summer to 25.5 °C in winter [28]. Approximately one gram of each poi was fermented at 25 °C for starch analysis, while 25 to 35 g of poi was fermented for mineral analysis, using a temperature-controlled incubator (VWR Model 1510E, Cornelius, OR 97113, USA). The samples were loosely capped during fermentation to enable gas exchange and were tightly capped post-fermentation. Each type of poi was fermented in parallel for 24 h and 48 h and was immediately frozen at −20 °C until the respective analysis.

### 2.3. Measurement of Resistant Starch (RS) and Non-Resistant Starch (NRS)

RS and NRS were analyzed in both fresh (non-fermented) and fermented poi using commercial Megazyme Resistant Starch Assay Kits (Cat# K-RSTAR, Bray Business Park, Bray, Co. Wicklow, A98 YV29, Ireland) according to the manufacturer’s protocol. In brief, 1 gm of each sample was mixed with pancreatic α-amylase (10 mg/mL) and amyloglucosidase (AMG, 3 U/mL) and incubated in a shaking water bath (200 strokes/min) for 18 h at 37 °C. After incubation, ethanol (99%) was added to each sample, vortexed, and centrifuged at 1500× *g* (approximately 3000 rpm) for 10 min. RS was recovered in the pellet, and the NRS was obtained from the supernatant.

For RS determination, the pellets were re-suspended and solubilized in 2 M potassium hydroxide and incubated in an ice bath on a platform rocker (Barnstead/Thermolyne Model M79735, Dubuque, IA 52001, USA) for 20 min. In total, 1.2 M sodium acetate buffer (pH 3.8) and AMG (3300 U/mL) were added to each tube and further incubated in a water bath at 50 °C for 30 min. During the incubation, samples were intermittently mixed by vortexing every 10 min. The samples were then centrifuged at 1500× *g* for 10 min, and aliquots of the undiluted supernatants were mixed with Glucose Determination Reagent (GOPOD; glucose oxidase plus peroxidase and 4-aminoantipyrine in p-hydroxybenzoic acid and sodium azide; 0.09% *w*/*v*) and incubated in a water bath at 50 °C for 20 min before analysis. Reagent blanks consisting of 100 mM sodium acetate buffer (pH 4.5) and GOPOD reagent and the D-glucose standards consisting of D-glucose (1 mg/mL) and GOPOD reagent were analyzed immediately without any further incubation.

For NRS determination, supernatants were diluted with 100 mM sodium acetate buffer (pH 4.5) and incubated with AMG solution (300 U/mL prepared in 100 mM sodium maleate buffer, pH 6.0) in a water bath at 50 °C for 20 min. GOPOD reagent was added to each sample and incubated in a shaking water bath for 20 min at 50 °C. In contrast, standards and reagent blanks, although mixed with GOPOD reagent, were not incubated further. For both the RS and NRS assays, the samples, standards, and the reagent blanks (200 uL of each) were transferred to a 96-well plate and analyzed in triplicate. The absorbance was measured at 520 nm using a Wallac Victor2 1420 Multilabel Counter (PerkinElmer Life Sciences, Boston, MA, USA). All values were combined from three independent assays for each poi at all different time points (*n* = 9).

### 2.4. Mineral and Moisture Analysis

Fresh and fermented poi samples were analyzed for mineral contents by the University of Hawai‘i Agriculture Diagnostic Service Center (ADSC, 1910 East-West Road, Sherman Laboratory 134, Honolulu, Hawai‘i 96822). The percentage of moisture content for each poi was determined during mineral analysis by measuring the differences between fresh and lyophilized dry poi weights. Each poi, at different fermentation time points, was analyzed in triplicate for each of the following minerals: boron (B), calcium (Ca), copper (Cu), iron (Fe), magnesium (Mg), manganese (Mn), molybdenum (Mo), phosphorus (P), potassium (K), sodium (Na), and zinc (Zn). The metals analyzed included arsenic (As), cadmium (Cd), chromium (Cr), nickel (Ni), lead (Pb), selenium (Se), and vanadium (Va).

The standard EPA 3050B method [29] was used to analyze minerals and metals with slight modifications [30]. In brief, 0.5 g of the lyophilized poi samples were digested with 3.5 mL of nitric acid (15.8 N) at 110 °C for 10 min. The samples were further mixed with 100 mL of double-distilled water, incubated on a shaker, filtered through Whatman filter paper #42, and analyzed on an Avio 560 Max Inductively Coupled Plasma-Optical Emission Spectrometer (ICP-OES, Perkin Elmer, WA, MA, USA).

### 2.5. DNA Extraction

DNA was extracted from fresh and fermented poi samples using the Qiagen DNeasy Blood and Tissue Kit (Cat# 69506, QIAGEN Strasse 1, 40724 Hilden, Germany) with a modified protocol. Approximately 150 mg of each fresh and fermented poi sample was mixed with a lysis buffer and proteinase K. The tubes were vortexed for 10 s and then incubated for 30 min at 56 °C in a shaking water bath, with a brief vortexing every 10 min during the incubation. After incubation, a second lysis buffer was added, the tubes were vortexed, and it was incubated for an additional 15 min in a shaking water bath at 56 °C. Samples were loaded into a DNeasy spin column after adding non-denatured ethanol (95%) and were centrifuged at 8000 rpm for 1 min. The flow-through was discarded and each column was washed with wash buffer and centrifuged for another minute at 8000 rpm. The second wash consisted of the same wash buffer and samples were centrifuged for 3 min at 13,000 rpm. The flow-through was discarded, and elution buffer was added to each spin column, which were transferred to a new microcentrifuge tube, incubated for one minute at room temperature, and centrifuged at 8000 rpm for one minute to elute the DNA. A second elution was performed using the same elution method. Concentration and purity of the DNA were determined using a NanoDrop spectrophotometer (ND-1000, NanoDrop Inc., 3411 Silverside Rd, Wilmington, DE 19810, USA). Each sample was extracted in triplicate. The V3–V4 regions of the 16S gene were amplified from the extracted DNA through Illumina MiSeq sequencing at the UH Mānoa Sequencing Facility, Advanced Studies in Genomics, Proteomics and Bioinformatics (ASGPB).

### 2.6. Library Preparation, 16S Sequencing, and Microbial Data Processing

The 16S rRNA gene libraries were prepared at the ASGPB UHM Sequencing Facility following the Illumina 16S Metagenomic Sequencing Library Preparation protocol, with slight modifications. Primers specific to the V3–V4 region of the 16S rRNA gene, appended with Illumina overhang adapters, were used. These primer sequences were adapted from Klindworth et al. [31], who evaluated general 16S ribosomal RNA gene PCR primers for both classical and next-generation sequencing-based diversity studies.

The gene-specific sequences of the primers are underlined.

16S Forward Primer:

5′-TCGTCGGCAGCGTCAGATGTGTATAAGAGACAGCCTACGGGNGGCWGCAG-3′

16S Reverse Primer:

5′-TCTCGTGGGCTCGGAGATGTGTATAAGAGACAGGACTACHVGGGTATCTAATCC-3′

Platinum Taq DNA Polymerase High Fidelity (Invitrogen/ThermoFisher, Carlsbad, CA, USA) was used to amplify the V3–V4 region in the first round of PCR. A denaturation temperature of 95 °C for 3 min, 35 cycles of 95 °C for 30 s, 55 °C for 30 s, and 68 °C for 30 s, followed by an extension of 68 °C for 5 min, was utilized in the first round of PCR. A negative PCR control (no DNA template) was included. The amplicons were checked on a 1% agarose gel and purified using Mag-Bind Total Pure NGS beads (Omega Bio-tek, Norcross, GA, USA). The second round of PCR used Nextera XT v2 indexes (Illumina, San Diego, CA, USA) with a denaturation temperature of 95 °C for 3 min, 8 cycles of 95 °C for 30 s, 55 °C for 30 s, and 68 °C for 30 s, followed by an extension of 68 °C for 5 min. After bead purification, the indexed libraries were quantified using the Quant-iT PicoGreen dsDNA Assay Kit (Invitrogen/ThermoFisher, Carlsbad, CA, USA), normalized, and pooled. The pooled library was run on a Bioanalyzer high-sensitivity DNA chip (Agilent, Santa Clara, CA, USA) to determine the average size. Sequencing was performed on an Illumina MiSeq to generate paired 300 bp reads.

The microbial sequences were analyzed and identified at the Bioinformatics Core, Department of Quantitative Health Sciences, University of Hawai‘i Cancer Center, John A. Burns School of Medicine, using the nf-core/ampliseq [32] pipeline (v2.1.0). In brief, the primers were first trimmed using Cutadapt [33] and then amplicon sequence variants (ASVs) were inferred using the DADA2 [34] workflow. In the DADA2 workflow, the sequence reads were truncated at 270 bp for the forward reads and 190 bp for the reversed reads and then filtered using a maximum expected error (MaxEE) of 5. After applying an error correction model, a list of unique sequences referred to as ASVs was obtained along with the number of their occurrences (reads) in each sample and negative controls. Potential chimeric sequences were removed before taxonomic classifications were assigned to each ASV using the RDP naive Bayesian classifier [35] against the reference database Silva [36] release 138.

### 2.7. Global Metabolomic Analysis

Metabolomics was conducted at the Fiehn laboratory, NIH West Coast Metabolomics Center. Global metabolites (targeted and untargeted) were analyzed by an automated linear exchange cold injection system gas chromatography time-of-flight mass spectrometer (ALEX-CIS GCTOF MS), as described previously [37,38,39,40,41]. In brief, 10 mg of each poi sample was homogenized and extracted with 1 mL of 3:3:2 acetonitrile (ACN)/isopropanol (IPA)/water by vortexing for 10 s and shaking for six minutes at 4 °C [37,38,39,40,41]. After centrifugation at 14,000 RCF (relative centrifugal force) for 2 min, the supernatant was aliquoted into 475 mL aliquots, dried, and stored until further analysis. Half of the dried sample was derivatized with 10 mL of 40 mg/mL of methoxyamine in pyridine and shaken at 30 °C for 1.5 h. In brief, 91 mL of *N*-methyl-*N*-(trimethylsilyl) trifluoroacetamide (MSTFA) + fatty acid methyl esters (FAMEs) were added to each sample and further shaken at 37 °C for 0.5 h to complete derivatization. Derivatized samples were injected on a 7890A gas chromatography (GC) coupled with a time-of-flight mass spectrometer (TOF; LECO Corporation, St. Joseph, MI) using a splitless method onto a RESTEK RTX-5SIL MS column with an Intergra-Guard at 275 °C with a helium flow of 1 mL/min^−1^. The GC oven was set at 50 °C for 1 min and then ramped to 330 °C at a rate of 20 °C/min and held for 5 min. The transfer line was set to 280 °C and the EI ion source was set to 250 °C. The MS parameters collect data from 85 *m*/*z* to 500 *m*/*z* at an acquisition rate of 17 spectra/s.

### 2.8. Statistical Data Analysis

Data for moisture, mineral, and metal contents are expressed as mean ± standard deviation of triplicate values. Statistical significance was assessed using GraphPad Prism 7.0. Data was analyzed using one-way ANOVA followed by Tukey’s the. *p*-values < 0.05 were considered to be significant.

Microbial ASV sequences were subjected to differential abundance analyses using the MicrobiomeAnalyst web-based platform (https://www.microbiomeanalyst.ca/; first accessed on 17 October 2024 ) [42,43,44]. Data were filtered by removing features with fewer than 4 counts and a prevalence of less than 20%. Low-abundance features based on the prevalence and low-variance features based on the inter-quantile range were also removed. The trimmed mean of M (TMM) values was used to normalize the data. Both alpha and beta diversity were calculated in MicrobiomeAnalyst. OTU counts were used to calculate alpha diversity, using a paired non-parametric test, while the Bray–Curtis index and PERMANOVA test were used to determine the beta diversity. For multiple comparisons, the false discovery rate correction method was applied as needed. Results with FDR < 0.05 were considered significant.

Metabolites (known and unknown) in fresh and fermented poi were analyzed by univariate and multivariate methods in MetaboAnalyst 6.0 (https://www.microbiomeanalyst.ca/; first accessed on 17 October 2024). Metabolite profiles from each poi sample at different fermentation time points were compared similarly. Data were normalized to the sample median, log10-transformed, and Pareto-scaled. Principal component analysis (PCA) was used to observe clustering trends and exclude outliers. A discriminant model was created by partial least squares discriminant analysis (PLS-DA) with 1000 permutation tests to check model validity and potential overfitting, visualized using a validation plot. Known metabolites in each category were used to build the PLS-DA models. After validation of the PLS-DA model, data were further analyzed by orthogonal partial least squares discriminant analysis (OPLS-DA) to identify discriminant metabolites and distinguish between each category at a false discovery rate (FDR) < 0.05.

In addition, Pearson’s correlation was used to assess relationships between normalized microbiome abundances and metabolite levels across five poi types at three fermentation time points.

## 3. Results

### 3.1. Nutrition Composition of Poi

Among the five commercial poi brands tested, nutrition labels were provided for only three brands (Aloha, Hanalei, and Taro), while the Pomai and Kokua brands did not have any nutrition information (Table 1). Fresh Pomai poi had the highest percentage of moisture among all brands and was significantly higher compared to Aloha and Kokua poi (Table 1, *p* < 0.05). The moisture content of fresh Kokua poi was lowest among all poi brands, although Kokua poi was only significantly lower than the Pomai and Taro brands (Table 1, *p* < 0.05). In all five brands of poi, the moisture content was not influenced by fermentation.

We also noted that the consistency and texture of all five poi brands were different, likely related to their moisture (water) contents. The consistency of fresh Hanalei and Pomai brand poi was thinner and there was more liquid than the other poi brands. Fresh Kokua brand poi had the thickest texture among all the poi. The consistency of the Aloha and Taro brand poi was between that of Hanalei and Pomai brand poi. We did not determine or compare the consistency of fermented poi.

### 3.2. Fermentation Had No Effect on Mineral Contents of Poi

Mineral contents of five commercial poi brands (Aloha, Hanalei, Kokua, Pomai, and Taro) were analyzed before (fresh, 0 h) and after fermentation (24 h and 48 h). The RDA values depicted in Table 2 are based on the recommended values by the National Academy of Sciences [45]. Although not statistically significant, total minerals were higher in Kokua > Taro > Hanalei > Pomai > Aloha (Table 2, *p* < 0.05). The amount of minerals in Aloha poi were in the order of K > P > Mg > Ca > Na > Fe > Zn > Mn > Cu > B > Mo, while those for the Hanalei and Pomai poi brands were in the order of K > P > Ca > Mg > Na > Fe > Zn > Cu > Mn > B > Mo (Table 2). The Kokua poi brand contained K > P > Ca > Mg > Na > Zn > Fe > Cu > Mn > B > Mo, while the Taro poi brand contained K > P > Mg > Ca > Na > Fe > Mn > Zn > Cu > B > Mo (Table 2).

It is noteworthy that based on the RDA values, all the commercial poi brands in our study were a rich source of minerals. Based on the lowest and highest mineral values among our commercial poi brands, the RDA amounts provided by poi for females are P: 28–42%, K: 37–65%, Ca: 16–20%, Mg: 46–71%, Fe: 34–66%, Mn: 100–304%, and Zn: 37–138% (Table 2). Similarly for males, the RDA amounts provided by poi brands in our study were P: 15–24%, K: 21–36%, Ca: 13–16%, Mg: 35–55%, Fe:76–150%, Mn: 100–304%, and Zn: 27–100%. Poi is very low in Na and provides about 1.4–4.5% of RDA (Table 2).

Overall, the P content of Kokua poi was significantly higher compared to Hanalei and Pomai poi (Table 2, *p* < 0.05). The potassium (K) contents of Kokua poi were significantly higher compared to Aloha poi at all time points (Table 2, *p* < 0.05). In contrast, K content in only fresh Taro poi was significantly higher compared to Aloha poi throughout the fermentation time points (Table 2, *p* < 0.05). Magnesium (Mg) contents of Aloha poi were significantly higher compared to Hanalei, Kokua, and Pomai poi at all time points, while Mg content in only fresh Taro poi was significantly higher compared to fresh Hanalei poi (Table 2, *p* < 0.05). Sodium (Na) contents of Kokua poi were significantly higher compared to Pomai poi at all time points (Table 2, *p* < 0.05).

Fresh Kokua poi had significantly higher Na content than both fresh Hanalei poi and Taro poi throughout fermentation time points, while Na content in Aloha poi (24 h) was significantly higher than Pomai poi at all time points (Table 2, *p* < 0.05). Zinc (Zn) contents were overall significantly higher in Kokua poi compared to Pomai, Taro, and Hanalei poi, and fresh Aloha poi was significantly higher compared to fresh Hanalei poi (Table 2, *p* < 0.05). Boron (B) contents were significantly lower in fresh Hanalei poi compared to Kokua poi across all time points (Table 2, *p* < 0.05). Fermented Kokua poi (48 h) had significantly higher B content compared to Hanalei and Pomai poi throughout fermentation time points, as well as in fermented Taro poi (48 h) (Table 2, *p* < 0.05). Ca, Fe, Mn, Cu, and Mo were not significantly different among any pois at all time points (Table 2, *p* < 0.05). 

Although individual poi demonstrated differences in their mineral contents, fermentation did not influence the levels of any minerals in our commercial poi brands (Table 2). No metals were detected in any of the poi brands in our study.

### 3.3. Fermentation Increases Resistant Starch (RS) Contents in Poi

**Figure 1:**
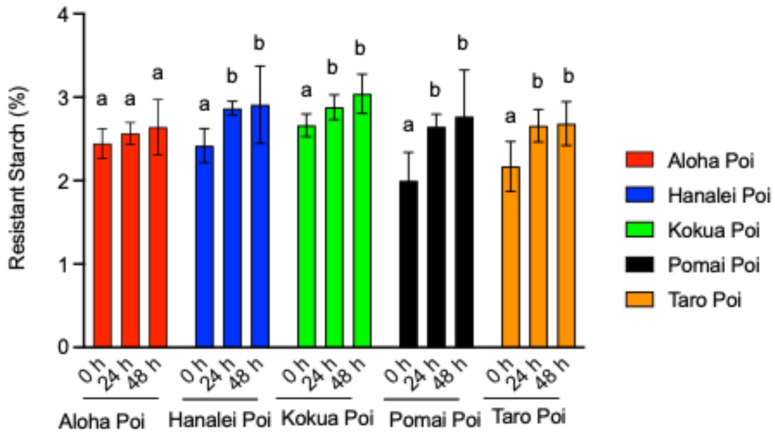
Effect of fermentation on resistant starch (RS) content (g/100 g of wet poi) for five local brands of individual poi—Aloha poi, Hanalei poi, Kokua poi, Pomai poi, and Taro poi—analyzed using GraphPad Prism 9.0. Values represent the mean ± S.E. (*n* = 9). Each sample was analyzed in triplicate; 0 h = fresh poi without fermentation, 24 h = 24 h after fermentation, and 48 h = 48 h after fermentation. ^a,b^ mean values with common letters do not differ (*p* < 0.05).

Changes in the RS content (g/per 100 g of wet poi) of the five poi brands were measured before (fresh, 0 h) as well as 24 h and 48 h after fermentation at 25 °C (Figure 1). Except for Aloha poi (Figure 1), the amount of RS in all other four poi brands was significantly increased by fermentation at 24 h and 48 h (Figure 1, *p* < 0.05). RS significantly increased by 18% to 20% in Hanalei poi (Figure 1, *p* < 0.05) and by about 8% to 14% in Kokua poi (Figure 1, *p* < 0.05) after 24 h and 48 h of fermentation, respectively, as compared to fresh poi. The highest increases in RS were noted in Pomai poi, demonstrating 32% to 38% (Figure 1), and Taro poi, displaying 22% to 23% (Figure 1), after 24 h and 48 h of fermentation, respectively (*p* < 0.05), as compared to fresh poi.

**Figure 2:**
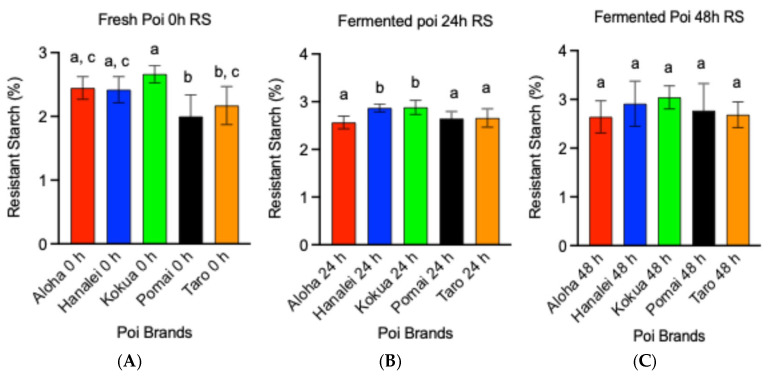
Effect of fermentation on resistant starch (RS) content (g/100 g of wet poi) of five local poi brands: (**A**) fresh poi, (**B**) after 24 h fermentation, and (**C**) after 48 h fermentation, analyzed using GraphPad Prism 9.0. Values represent the mean ± S.E. (*n* = 9). Each sample was analyzed in triplicate. ^a,b,c^ mean values with common letters do not differ (*p* < 0.05).

Among the five fresh poi brands (0 h fermentation), the RS content of Kokua poi was 33% higher compared to Pomai poi and 23% higher compared to Taro poi, which was significant (Figure 2A, *p* < 0.05). A similar comparison of RS contents of all five poi brands after 24 h of fermentation indicated that the RS content of Aloha poi was significantly lower by 13% and 3% compared to Hanalei and Kokua poi, respectively (Figure 2B, *p* < 0.05). The RS content of Hanalei and Kokua poi was about 8% to 9% higher compared to Pomai and Taro poi after 24 h of fermentation (Figure 2B, *p* < 0.05). Figure 2C demonstrates that the RS content of all five poi brands after 48 h of fermentation was not significantly different (*p* < 0.05).

### 3.4. Fermentation Increases Non-Resistant Starch (NRS) Contents of Poi

**Figure 3:**
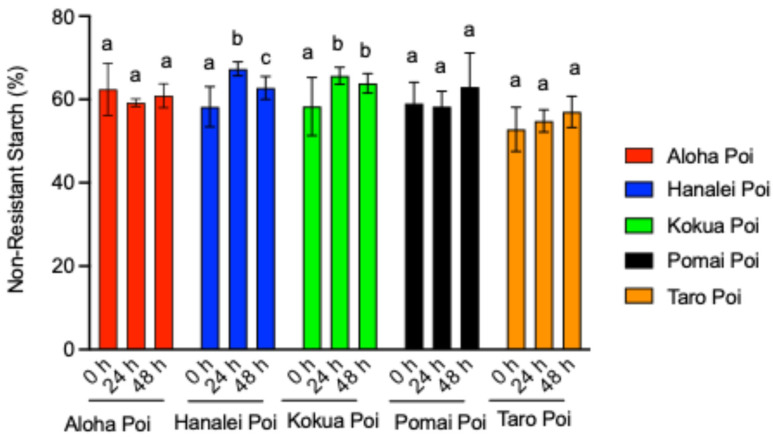
Effect of fermentation on non-resistant starch (NRS) content (g/100 g of wet poi) in five brands of locally available individual poi—Aloha poi, Hanalei poi, Kokua poi, Pomai poi, and Taro poi—analyzed using GraphPad Prism 9.0. Values represent the mean ± S.E. (*n* = 9). Each sample was analyzed in triplicate; 0 h = fresh poi without fermentation, 24 h = 24 h fermentation, and 48 h = 48 h fermentation. ^a,b,c^ mean values with common letters do not differ (*p* < 0.05).

Figure 2 depicts the changes in NRS content (g/per 100 g of wet poi) for five different commercial poi brands, measured before (fresh, 0 h) as well as after 24 h and 48 h of fermentation at 25 °C. The NRS contents of Aloha poi were unaffected by fermentation (Figure 3). As compared to fresh poi (0 h), fermentation significantly increased NRS in Hanalei poi by 15% and 7% after 24 h and 48 h of fermentation, respectively (Figure 3, *p* < 0.05). After 48 h of fermentation, the NRS in Hanalei poi was 7% lower than the 24 h fermented Hanalei poi (Figure 3, *p* < 0.05). In Kokua poi, fermentation for 24 h and 48 h also significantly increased the NRS contents by 12% and 9%, respectively, as compared to fresh Kokua poi (0 h, Figure 3, *p* < 0.05). The NRS contents of the Pomai and Taro poi brands were unaffected by fermentation (Figure 3, *p* < 0.05).

**Figure 4:**
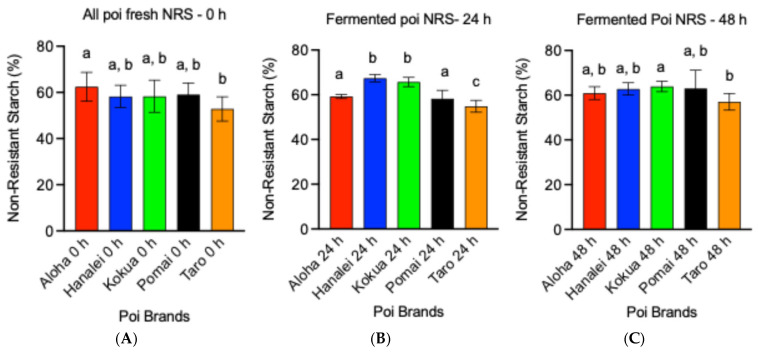
Effect of fermentation on non-resistant starch (NRS) content (g/100 g of wet poi) of five local poi brands: (**A**) fresh poi, (**B**) after 24 h fermentation, and (**C**) after 48 h fermentation, analyzed using GraphPad Prism 9.0. Values represent the mean ± S.E. (*n* = 9). Each sample was analyzed in triplicate. ^a,b,c^ mean values with common letters do not differ (*p* < 0.05).

Figure 4 illustrates the NRS contents of all five poi brands at different stages of fermentation, fresh (Figure 4A), 24 h after fermentation (Figure 4B), and 48 h after fermentation (Figure 4C). Taro brand poi contained significantly lower NRS (18% lower) compared to Aloha poi (Figure 4A, *p* < 0.05). The amount of NRS in Hanalei, Kokua, and Pomai poi was comparable with Aloha poi and was not significantly different than Taro poi (Figure 4A, *p* < 0.05). Comparing all poi brands after 24 h of fermentation, the Taro brand had a significantly lower amount of NRS compared to Aloha (7.4% lower), Hanalei (18.6% lower), Kokua (16.5% lower), and Pomai poi (5.9% lower, Figure 4B, *p* < 0.05). After 48 h of fermentation, the amount of NRS in Taro poi was still significantly lower compared to Kokua poi by 12% (Figure 4C, *p* < 0.05). The NRS amounts of all other poi were comparable after 48 h of fermentation (Figure 4C, *p* < 0.05).

### 3.5. Alpha and Beta Diversity for Individual Poi

**Figure 5:**
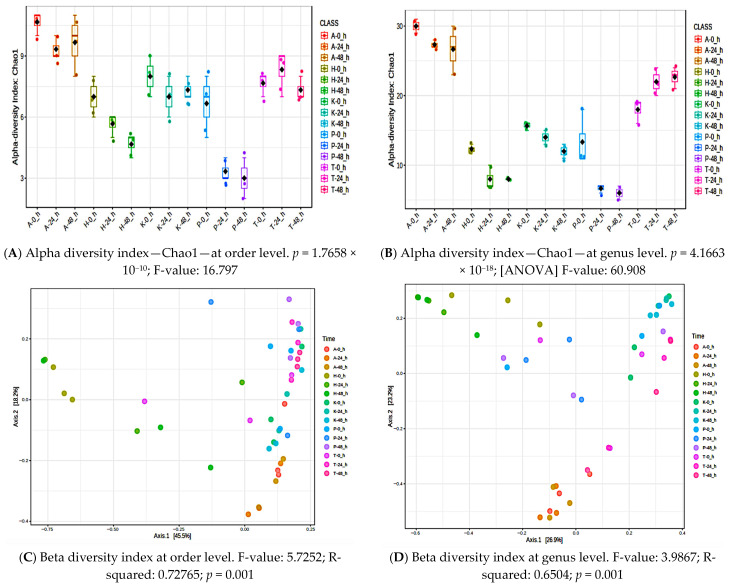
Alpha and beta diversity comparing all five poi types. (**A**) Alpha diversity index—Chao1—at order level. (**B**) Alpha diversity index—Chao1—at genus level. (**C**) Beta diversity index at order level, and (**D**) beta diversity index at genus level.

Richness and diversity of bacterial communities were evaluated by calculating three alpha diversity indices, Chao1 (richness), Shannon (richness and evenness), and Simpson (dominance), for each poi types at 0 h (fresh) and after 24 h and 48 h of fermentation (Table 3). The alpha and beta diversity at the order level were significant only for the Pomai poi (Table 3, *p* < 0.05 at FDR < 0.05). Higher F-values for the Pomia poi indicate that between-group variation in bacterial community composition is larger relative to within-group variation. The microbial communities were significantly different between fresh and fermented Pomai poi as indicated by the beta diversity indices (Table 3, *p* < 0.05 at FDR < 0.05). Figure 5 depicts the alpha and beta diversities at the order and genus levels for all poi brands. The alpha diversity Chao1 index as well as the beta diversity index at the order (Figure 5A and Figure 5C, respectively) and genus (Figure 5B and Figure 5D, respectively) levels were significantly different among different poi brands (*p* < 0.05).

### 3.6. Fermentation Redistributes the Relative Abundance and Microbial Diversity of Poi

Subsequently, 16S rRNA gene profiles were used to determine the microbial communities in fresh (0 h) and fermented poi (24 h and 48 h) samples. To identify the effects of 24 h and 48 h fermentation on microbial abundances in poi, we compared two groups at a time (24 h vs. 0 h, 48 h vs. 0 h, and 48 h vs. 24 h) using EdgeR (MicrobiomeAnalyst, accessed on 3 April 2024 https://www.microbiomeanalyst.ca/MicrobiomeAnalyst/home.xhtml; https://github.com/xia-lab/MicrobiomeAnalystR). Significant findings are presented in Table 4 and Table 5.

**Figure 6:**
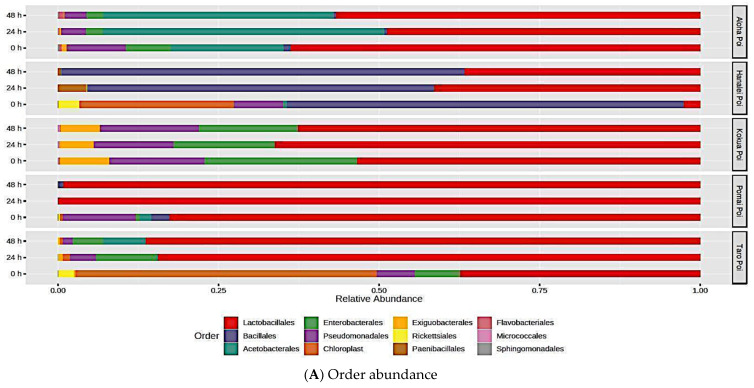

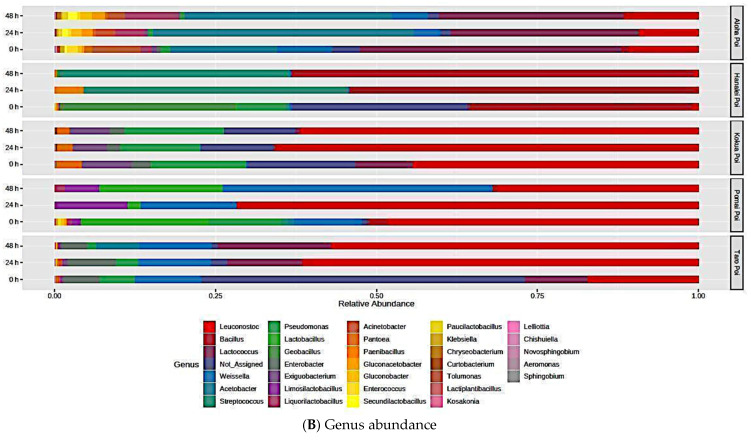
Microbial relative abundance of fresh and fermented poi. The effect of fermentation on microbial relative abundance in five different commercial brands of poi at the order level, (**A**), and at the genus level, (**B**).

At the order level, *Lactobacillales* was the most abundant of microbes in all types of poi except for the fresh Hanalei brand (0 h fermentation, Figure 6A). Compared to fresh poi, fermentation at 24 h and 48 h significantly increased *Acetobacterales* by more than 2.5-fold in Aloha poi (Figure 6A, Table 4, *p* < 0.05, FDR < 0.05). Fresh (0 h fermentation) Hanalei poi was mostly populated by *Bacillales* followed by *Chloroplast*, *Pseudomonadales*, *Rickettciales*, *Lactobacillales*, and *Acetobacterales*. Fermentation of Hanalei up to 48 h significantly increased the abundances of *Bacillales*, *Lactobacillales*, and *Paenibacillales* by 5- to 9-fold, while *Rickettsiales* was reduced by 3- to 6-fold, compared to fresh (0 h) poi (Figure 6A, Table 4, *p* < 0.05, FDR < 0.05). In contrast, *Chloroplast* was significantly reduced by 4-fold after 48 h of fermentation as compared to the fresh Hanalei poi (Table 4, *p* < 0.05, FDR < 0.05). Interestingly, fermentation did not significantly affect the microbial diversity of Kokua poi except for a 4-fold increase in *Staphylococcales* bacteria after 24 h of fermentation as compared to fresh Kokua poi (Table 4, *p* < 0.05, FDR < 0.05). Kokua poi was mostly populated by *Lactobacillales* followed by *Acetobacterales*, *Bacillales*, *Exiguobacterales*, and *Pseudomonales* (Figure 6A). In Pomai poi, *Lactobacillales* was the most predominant bacteria after 24 h and 48 h of fermentation (5- to 4-fold increases, respectively, Table 4, *p* < 0.05, FDR < 0.05) in contrast to the fresh Pomai poi, which contained 85% *Lactobacillales,* with small amounts of *Bacillales*, *Acetobacterales*, *Pseudomonadales*, and *Chloroplast* (Figure 6A). As noted in Table 4, *Pseudomonadales* was significantly reduced by 5-fold in Pomai poi after fermentation at 24 h and 48 h, while *Acetobacterales* was significantly reduced by 6.7-fold after 48 h of fermentation compared to fresh Pomai poi (*p* < 0.05, FDR < 0.05). Fermentation also changed the predominant bacteria in the Taro poi brand. *Chloroplast,* which was abundant in fresh Taro poi (0 h fermentation), was significantly reduced by 7-fold after 24 h and 48 h of fermentation (Table 4, *p* < 0.05, FDR < 0.05). Similarly, *Rickettsiales* and *Veillonellales_Selenomon* bacteria were reduced by 6- to 7-fold after fermentation compared to fresh Taro brand poi (Table 4, *p* < 0.05, FDR < 0.05). Additionally, a significant reduction in *Bacillales* by 7-fold and increases in *Acetobacterales* (6.7-fold) and *Lactobacillales* (3.2-fold) were noted after 48 h of fermentation as compared to fresh Taro poi (Table 4, *p* < 0.05, FDR < 0.05).

At the genus level, *Leuconostoc*, *Bacillus*, and *Lactococcus* were the most abundant types of microbes among all types of poi (Figure 6B). Fermentation for 48 h seemed to significantly reduce *Erwinia* and increase *Lacticaseibacillus*, *Liquorilactobacillus*, *Ameyamaea*, and *Gluconobacter*, as compared to fresh Aloha poi (Table 5, *p* < 0.05, FDR < 0.05). As noted in Table 5, *Streptococcus*, *Bacillus*, *Leuconostoc*, and *Paenibacillus* were significantly increased by 11- to 3-fold, while *Geobacillus* was reduced by 3- to 4-fold in fermented Hanalei poi as compared to fresh Hanalei poi (Table 5, *p* < 0.05, FDR < 0.05). Fermentation did not affect bacterial abundance at the genus level in Kokua, Pomai, and Taro poi brands (Table 5).

### 3.7. Global Metabolite Signatures of Five Local Brands of Fresh Poi

A total of 431 metabolites tentatively assigned as global metabolites (159 known and 272 unknown) were detected in all five types of poi brands (fresh and fermented poi). All fresh poi contained several free sugars, sugar acids, sugar alcohols, organic acids, fatty acids, two flavonoids (epicatechin and catechin), vitamin E (α and γ tocopherols), and a total of 18 amino acids that included nine essential and nine non-essential amino acids (Table S1). A total of 85 metabolites tentatively assigned as global metabolites (46 known and 39 unknown) were significantly different among the five brands of fresh poi as determined by a one-way ANOVA Fisher’s least significant difference (LSD) post hoc test (Table S2, *p* < 0.05, FDR < 0.05).

**Figure 7:**
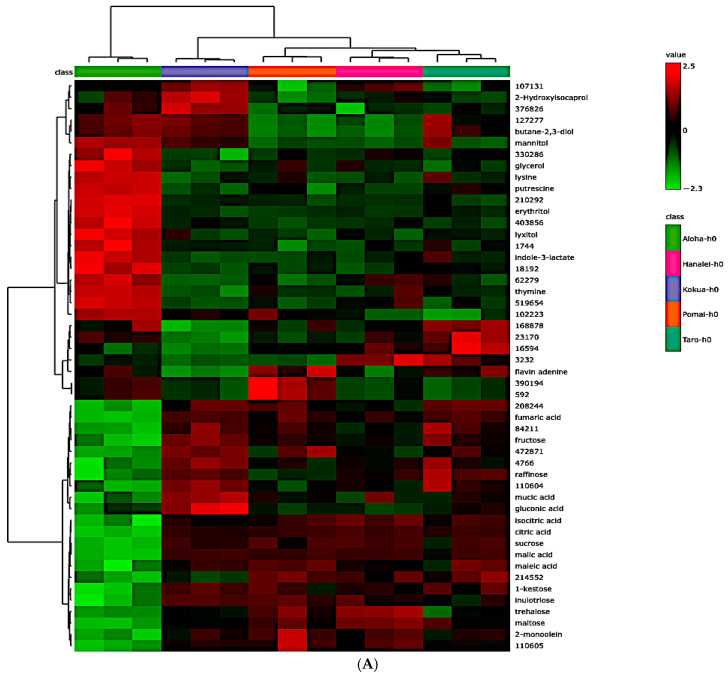

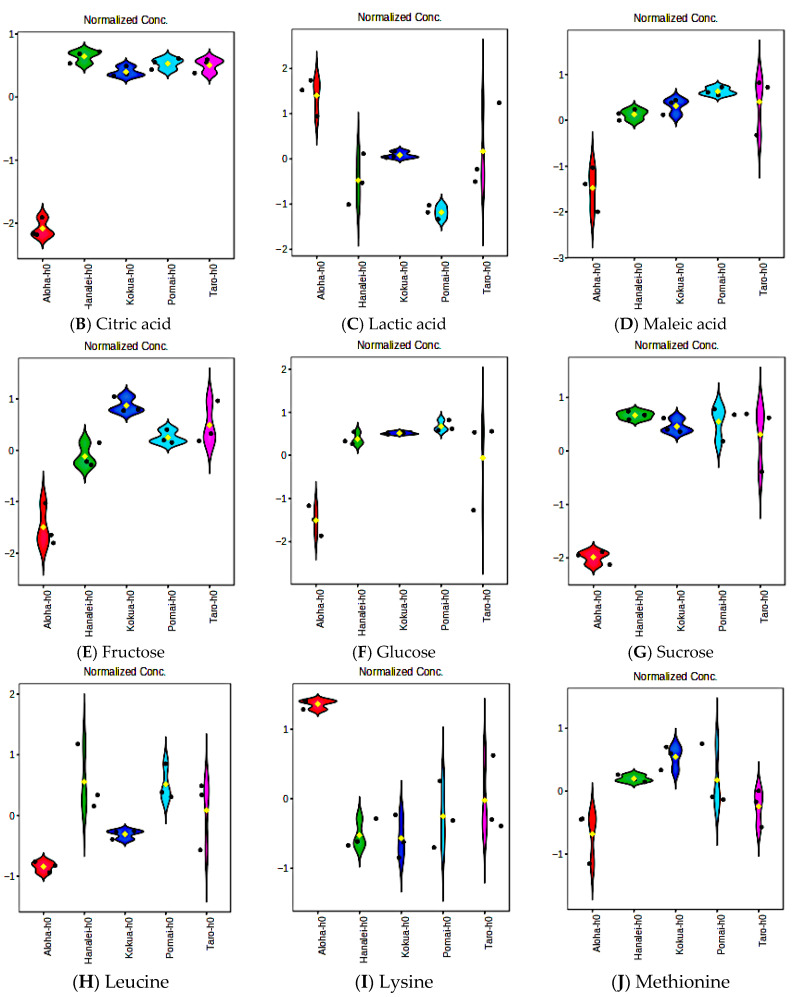
Metabolite signatures in fresh poi. (**A**) Heatmap of the top 50 significant known metabolites. Selected violin plots (Metaboanalyst 6.0) for significantly different metabolites in fresh poi: (**B**) citric acid, (**C**) lactic acid, (**D**) maleic acid, (**E**) fructose, (**F**) glucose, (**G**) sucrose, (**H**) leucine, (**I**) lysine, and (**J**) methionine (*n* = 3, *p* < 0.05).

When 159 of the known global metabolites were subjected to univariate and multivariate analyses, 63 of these known metabolites were significantly different among the five brands of fresh poi (Table S3, *p* < 0.05, FDR < 0.05). A heatmap of the significantly different top 50 known metabolites, as well as violin plots of significantly different amino acids, sugars, and organic acids in five brands of fresh poi, are depicted in Figure 7 (*p* < 0.05, FDR < 0.05). As noted in the Figure 7A heatmaps, the metabolite pattern in fresh Aloha poi is distinctly different than Hanalei, Kokua, Pomai, and Taro poi brands. The concentration of organic acids such as citric acid, maleic acid, and lactic acid, as well as major free sugars such as sucrose, lactose, glucose, and fructose, were significantly different in fresh poi brands (Figure 7B–G, *p* < 0.05). Among the 18 amino acids that were detected, the amount of leucine, lysine, and methionine was significantly different in fresh poi brands (Figure 7H, Figure 7I, and Figure 7J, respectively, *p* < 0.05).

### 3.8. Comparing Global Metabolites in Five Local Poi Brands After 24 h and 48 h of Fermentation

**Figure 8:**
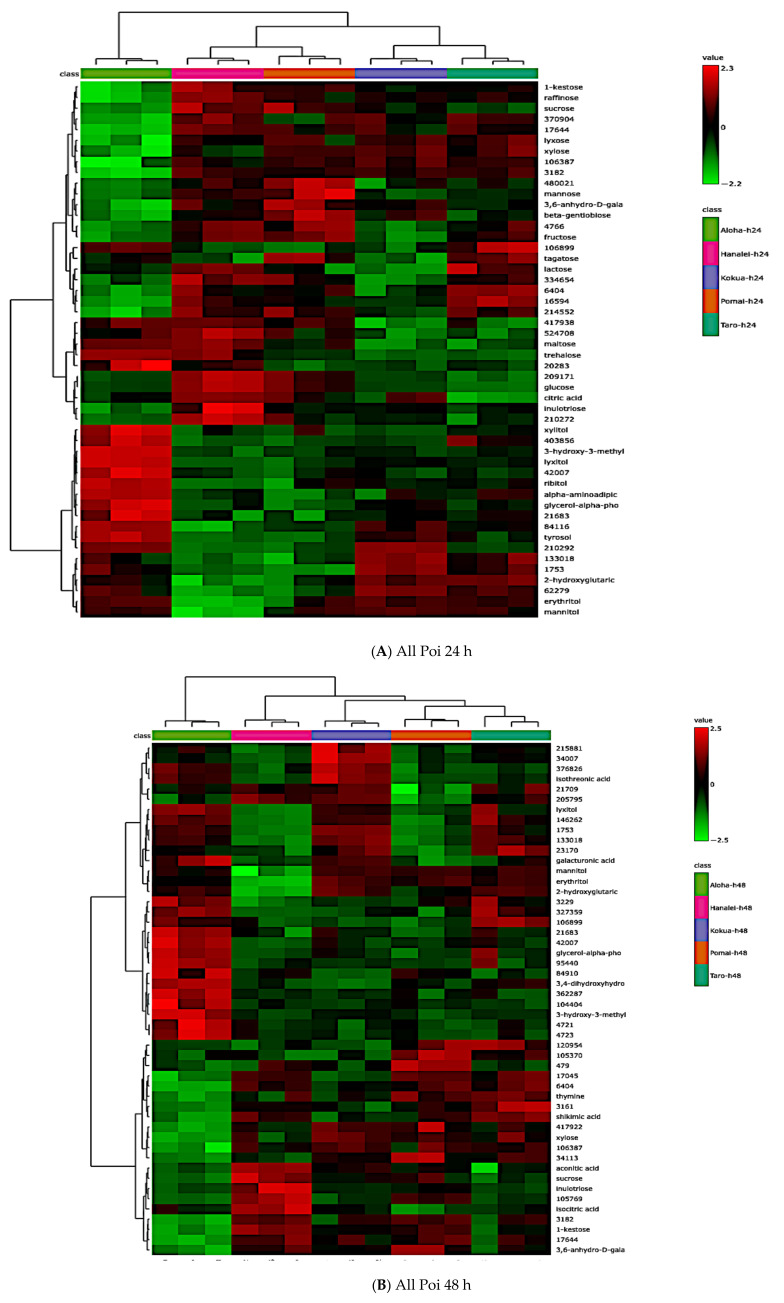

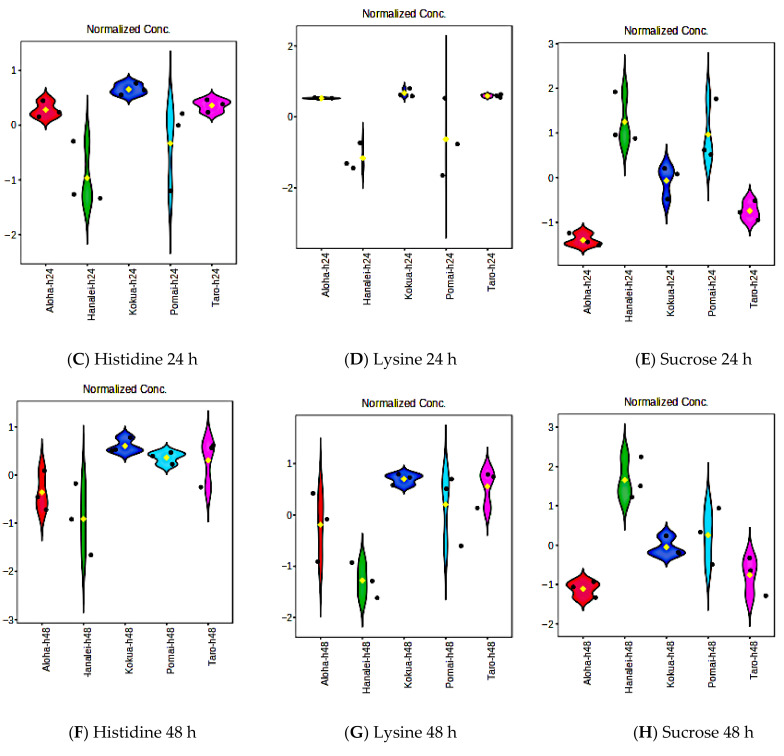
Hierarchical cluster analysis (HCA) of the top 50 significant metabolites comparing fermentation effects in all poi 24 h after fermentation (**A**) and 48 h after fermentation (**B**). Selected violin plots comparing significant differences in amino acids and sugars after 24 h of fermentation (**C**–**E**) and 48 h of fermentation (**F**–**H**), respectively (*n* = 3, *p* < 0.05).

After 24 h of fermentation, 103 global metabolites (43 known and 60 unknown) were significantly different in all five poi brands (Table S9, *p* < 0.05, FDR < 0.05). Similarly, after 48 h of fermentation, 86 global metabolites (36 known and 50 unknown) were significantly different in all five poi brands (Table S10, *p* < 0.05, FDR < 0.05). Figure 8 depicts the hierarchical clustering of all five poi brands after 24 h of fermentation (Figure 8A) and 48 h of fermentation (Figure 8B). Significant changes in histidine, lysine, and sucrose after 24 h and 48 h of fermentation are demonstrated in Figure 8C, Figure 8D, Figure 8E, Figure 8F, Figure 8G, and Figure 8H, respectively (*p* < 0.05).

### 3.9. Effect of Fermentation on Global Metabolite Signatures of Individual Poi Brands

**Figure 9:**
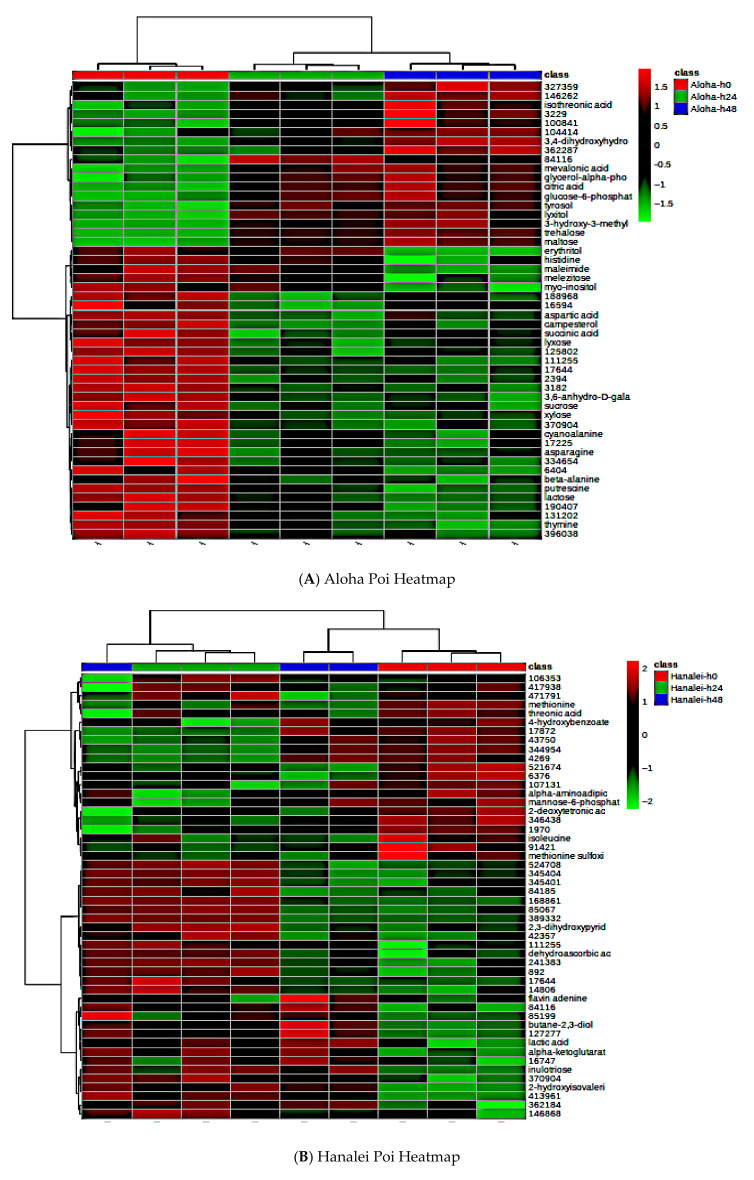

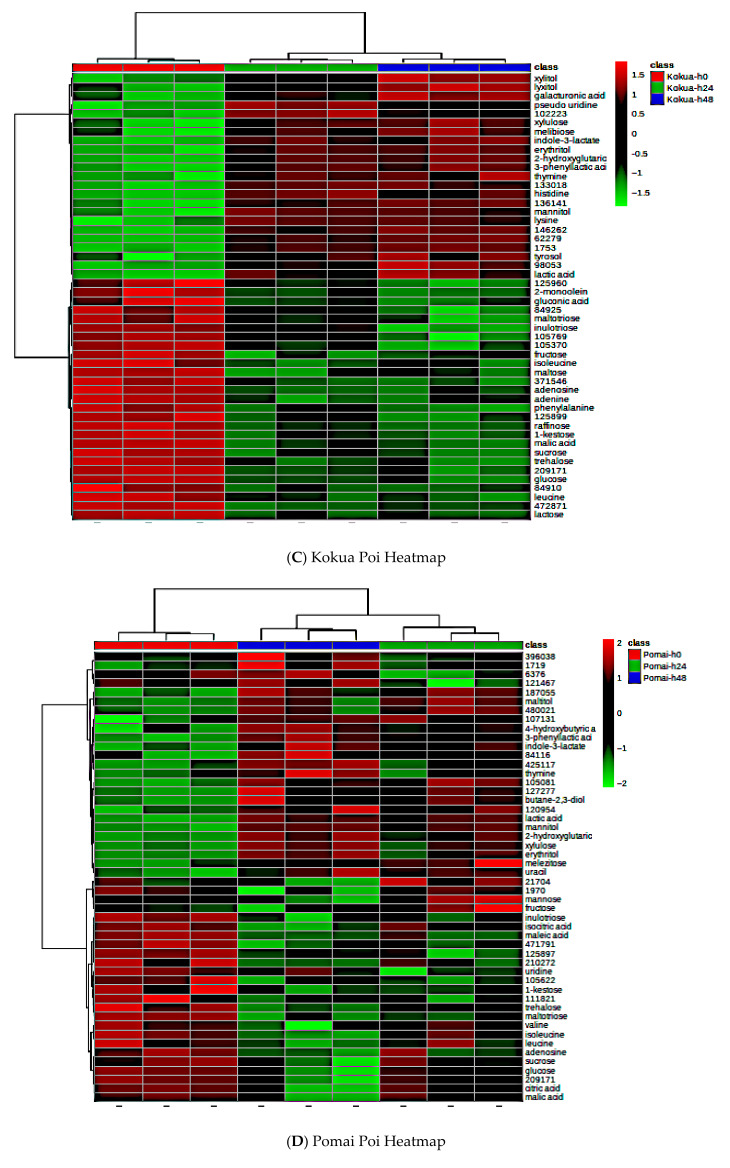

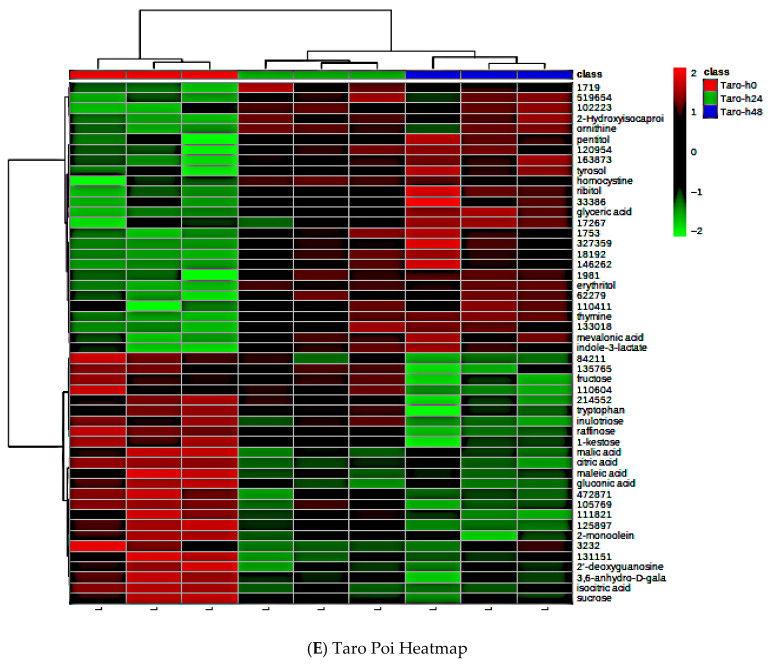

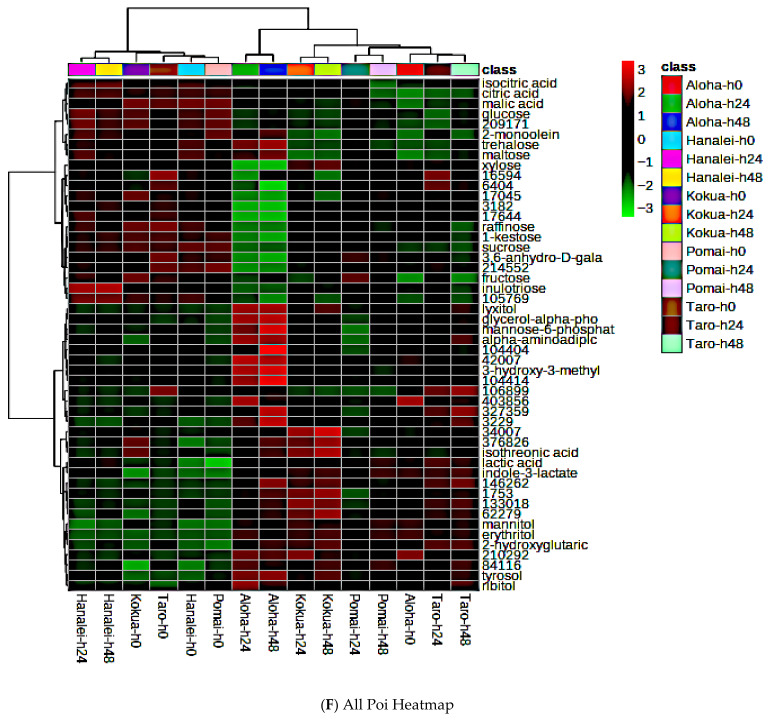
Hierarchical cluster analysis (HCA) of the top 50 significant metabolites demonstrating fermentation effects in poi (*n* = 3, *p* < 0.05). (**A**) Aloha poi, (**B**) Hanalei poi, (**C**) Kokua poi, (**D**) Pomai poi, (**D**) Taro poi, and (**E**) heatmap comparing all poi brands. (**F**) Heatmap comparing all poi brands.

A shift in the global metabolite (known and unknown) pattern was evident upon fermentation in four poi brands (Figure 9). Univariate analysis indicated that 54 global metabolites (32 known and 22 unknown) were significantly different in fresh Aloha poi compared to Aloha poi fermented for 24 h and 48 h (Figure 9A, *p* < 0.05, FDR < 0.05, Table S4). Similarly, fermentation significantly affected 73 global metabolites (44 known and 29 unknown) in Kokua poi (Figure 9C, *p* < 0.05, FDR < 0.05, Table S5), 2 known global metabolites in Pomai poi (Figure 9D, *p* < 0.05, FDR < 0.05, Table S6) and a total of 7 (6 known and 1 unknown) global metabolites in Taro brand poi (Figure 9E, *p* < 0.05, FDR < 0.05, Table S7). Interestingly, fermentation did not affect the metabolite abundances in Hanalei poi (Figure 9B). A total of 243 global metabolites (104 known and 139 unknown) were significantly different when all five poi brands were compared (Figure 9F, *p* < 0.05, Table S8).

**Figure 10:**
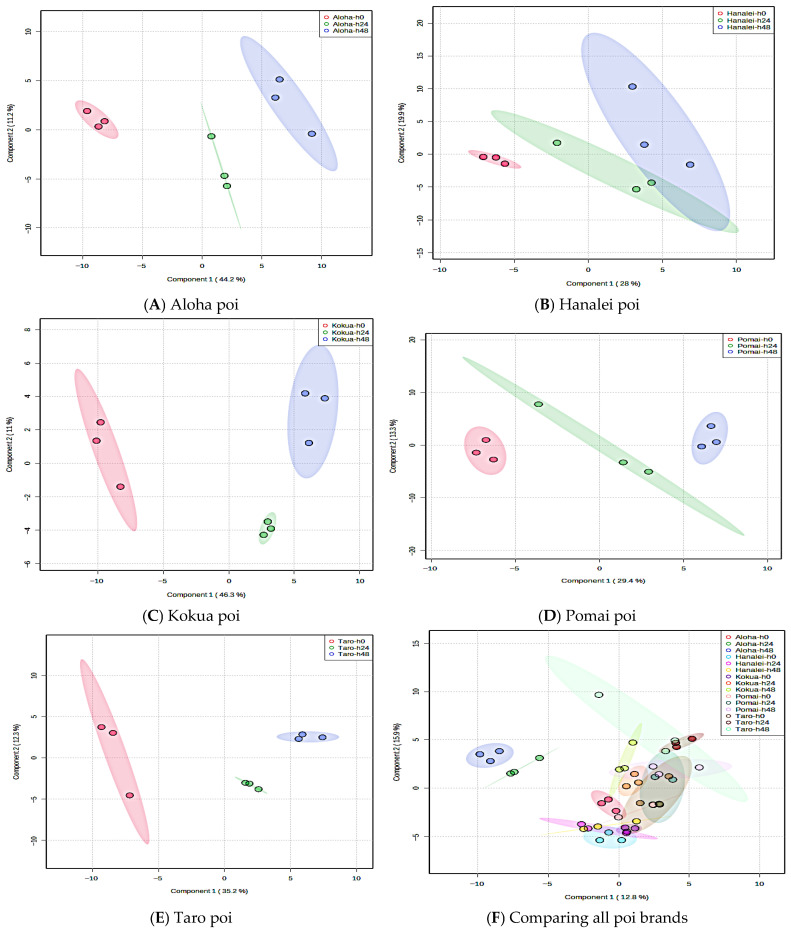
PLS-DA score plots for five poi brands. (**A**): Aloha poi (*n* = 3), (**B**): Hanalei poi (*n* = 3), (**C**): Kokua poi (*n* = 3), (**D**): Pomai poi (*n* = 3), (**E**): Taro poi (*n* = 3), and (**F**): all poi brands (*n* = 3 for each poi and at each fermentation time).

The PLS-DA score plots for individual poi brands and fermentation times are represented in Figure 10. After fermentation, there was a trend of decreasing concentration in the free sugars and an increasing trend in some organic acids such as citric acid, maleic acid, and lactic acid (Tables S1–S8).

### 3.10. Fermentation Increases Amino Acid Contents of Poi but Does Not Affect Essential Fatty Acids, Vitamin E, or Flavanols

**Figure 11:**
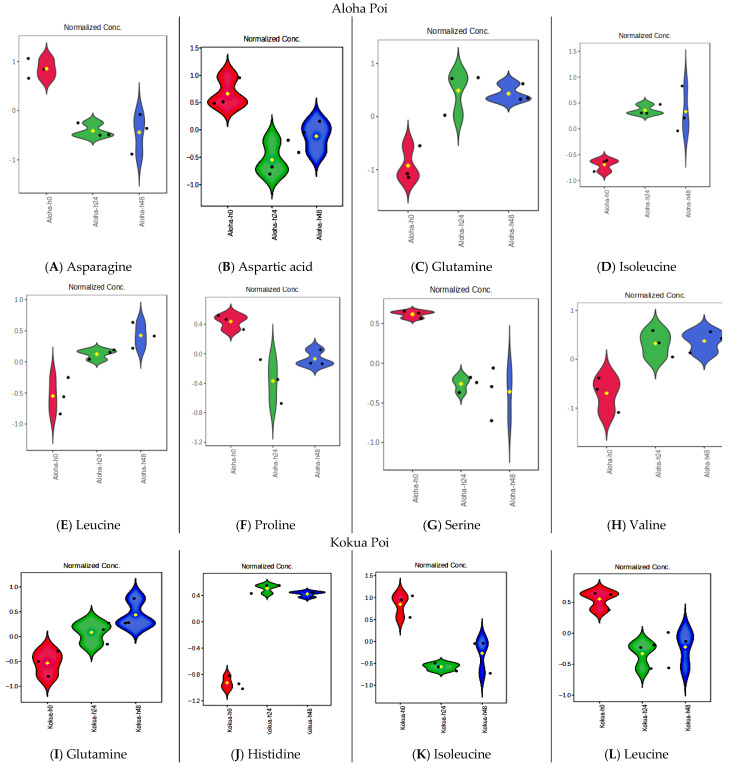

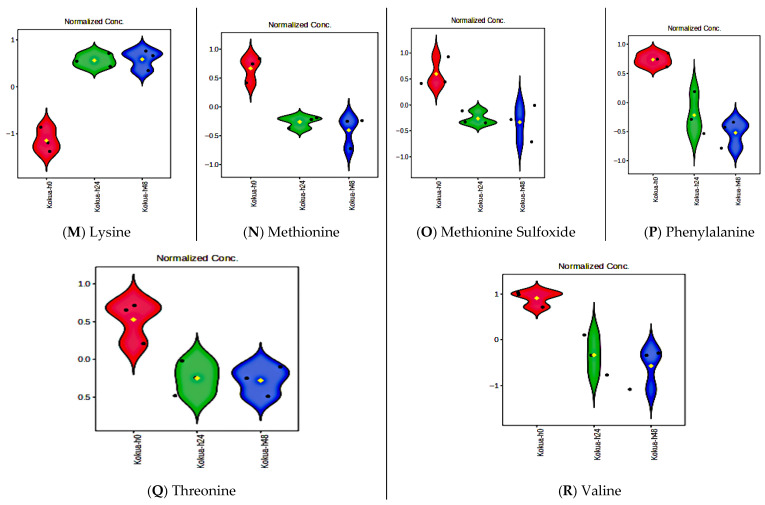
Significant effects of fermentation in amino acid contents of Aloha (**A**–**H**) and Kokua poi ((**I**–**R**); *n* = 3, *p* < 0.05). Relative abundances of amino acid contents in Hanalei, Pomai, or Taro poi were unaffected by fermentation. Red color = fresh (o h) poi, green color = 24 h fermentation and blue color = 48 h fermentation.

Among the 20 amino acids, a total of 18 amino acids were identified in all poi brands, while arginine and cysteine were absent (Table S1). All of the nine essential amino acids, viz. histidine, isoleucine, leucine, lysine, methionine, phenylalanine, threonine, tryptophan, and valine were detected in all poi brands (Table S1). Glutamine, isoleucine, leucine, and valine were significantly increased in Aloha poi after 24 h and 48 h of fermentation as compared to fresh (0 h) Aloha poi (Figure 11C, Figure 11D, Figure 11E, and Figure 11H, respectively, *p* < 0.05, FDR < 0.05). Asparagine, aspartic acid, proline, and serine were significantly reduced in Aloha poi after 24 h and 48 h of fermentation as compared to fresh (0 h) Aloha poi (Figure 11A, Figure 11B, Figure 11F, and Figure 11G, respectively, *p* < 0.05, FDR < 0.05).

In Kokua poi, glutamine, histidine, and lysine were significantly increased after 24 and 48 h of fermentation as compared to fresh Kokua poi (Figure 11I, Figure 11J, and Figure 11M, respectively, *p* < 0.05, FDR < 0.05). In contrast, isoleucine, leucine, methionine, methionine sulfoxide, phenylalanine, threonine, and valine were significantly reduced in fermented Kokua poi as compared to fresh Kokua poi (Figure 11K, Figure 11L, Figure 11N, Figure 11O, Figure 11P, Figure 11Q, and Figure 11R, respectively, *p* < 0.05, FDR < 0.05). In contrast to Aloha and Kokua poi, fermentation had no effect on the 18 amino acids present in Hanalei, Pomai, or Taro poi. Similarly, fatty acids such as oleic acid, linolenic acid and linolic acid, α- and γ-tocopherols, as well as flavanols such as catechin and epicatechin were unaffected by fermentation.

### 3.11. Pearson Correlation Analysis of Fermenting Bacteria and Global Metabolites in Individual Poi Brands

**Figure 12:**
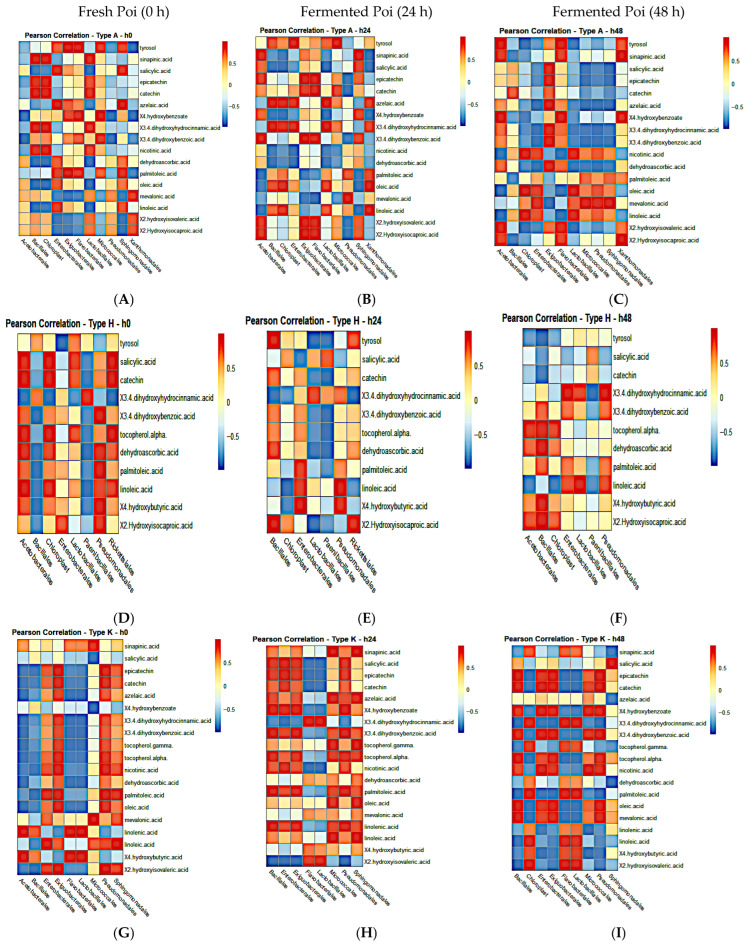

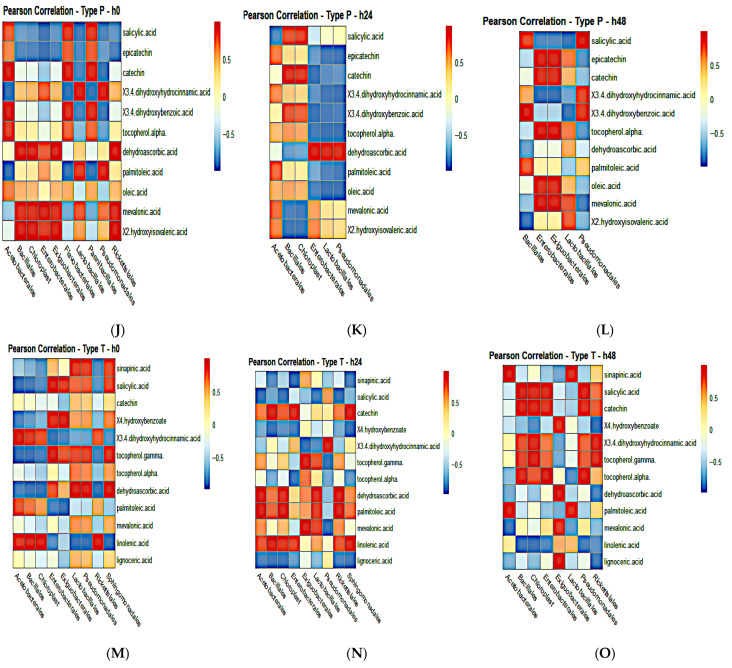
Pearson correlation between fermenting bacteria and global metabolites. (**A**) Aloha poi (0 h), (**B**) Aloha poi (24 h), (**C**) Aloha poi (48 h), (**D**) Hanalei poi (0 h), (**E**) Hanalei poi (24 h), (**F**) Hanalei poi (48 h), (**G**) Kokua poi (0 h), (**H**) Kokua poi (24 h), (**I**) Kokua poi (48 h), (**J**) Pomai poi (0 h), (**K**) Pomai poi (24 h), (**L**) Pomai poi (48 h), (**M**) Taro poi (0 h), (**N**) Taro poi (24 h), (**O**) Taro poi (48 h).

Taxa abundance at the order level and abundance profiles of the poi metabolites were subjected to Pearson correlation to identify the bacterial taxa responsible for changes in poi global metabolites. It was difficult to identify any particular bacteria that were consistently associated with significant changes in metabolite abundances. Overall, 11 to 19 bacterial types were significantly associated with abundances of 5 to 11 metabolites in all types of poi brands and at varying fermentation times (Figure 12A–E, *p* < 0.05, FDR < 0.05).

## 4. Discussion

To our knowledge, this is the first comprehensive evaluation of minerals, metabolites, and bacterial contents of commercial poi brands from Hawai‘i. Poi is prepared by mashing cooked taro corns to the desired consistency (mostly thick pudding). Poi is considered a superfood since it is almost fat-free, high in fiber, and has essential vitamins and minerals, including calcium, iron, and phosphorus. Although the amount of total protein in poi is 1% or less (based on nutritional labels on poi) [46], our study is the first to scientifically report the presence of 18 amino acids, including all of the nine essential amino acids in all five poi brands [24]. It is no wonder then that poi is one of the first foods introduced to babies in Hawaiʻi, specifically among Native Hawaiians, Pacific Islanders, and Filipinos [17,47,48]. Traditionally, poi has been used to treat infants with food allergies, failure-to-thrive, as well as digestive disorders like celiac disease among adults [14].

Nutritional composition and mineral contents of poi can be influenced by multiple factors, including the variety or cultivars of taro, the maturity of the tubers at harvest, the geographical location of taro farms, agricultural practices, as well as poi preparation methods [13,49,50]. The nutritional labels on all five poi brands reported similar amounts of carbohydrates, proteins, fats, minerals, and vitamins. Our independent analysis indicated that the moisture contents in all five poi brands ranged from 76% to 84%, which is similar to that reported by the USDA (71.6%) [46]. The mineral contents for all poi brands were higher in our study compared to those reported on the nutritional labels and the USDA’s comprehensive food composition data [46]. It should be noted that most of the nutritional information of poi on the USDA website is from 1984 to 2009 and has not been updated since then [46]. For example, nutritional labels on poi packages and the USDA website report that poi provides 2% of RDA for Ca, while our data suggests that poi can provide 13–20% of RDA for Ca. Additionally, differences in mineral values (nutritional labels vs. our study) may possibly arise due to differences in analytical methods. The mineral contents of taro from independent studies are also higher than those listed by the USDA and are comparable to those found in the poi brands analyzed in our study [49,51]. For example, the most abundant mineral identified in our commercial poi brands was K, which was also the most abundant mineral identified in cooked taro [49]. All poi brands in our study are a good source of minerals and specifically higher in K and P followed by Ca and Mg. All poi brands were a good source of Fe, providing up to 34–66% of RDA values for women and 76–150% for men. Similarly, all poi brands contain Zn and can provide 37–138% of RDA values for women and 27–100% for men.

Interestingly, fermentation did not change the mineral contents of any poi brands. Studies have indicated that the effects of fermentation on the changes in the mineral contents of foods are not uniform and may be dependent upon specific food being fermented, bacterial strains, and/or the fermentation conditions, such as the duration of fermentation and temperature. The relative concentration of minerals may sometimes appear to increase due to fermentation, probably due to a loss of dry matter, since bacteria may consume carbohydrates and proteins during fermentation. Sometimes, minerals can also be lost during fermentation through leaching or consumption by microorganisms. However, the bioavailability of minerals (absorption in the body) may be increased due to fermentation-based changes in the food matrix. Fermentation may not necessarily change their total content since minerals are not created/synthesized during fermentation. Antinutrients like phytates and oxalates in plant-based foods can bind to minerals (such as iron, zinc, and calcium) and inhibit their absorption. Phytase produced by LAB can break down these antinutrients, releasing the chelated minerals and increasing their absorption. Fermentation can also change the chemical forms of minerals for easy absorption.

Micronutrient “inadequacies” (intake below the daily average requirement but above the deficiency level) are prevalent in the United States and worldwide [52,53], which may lead to “hidden hunger”, a condition characterized by micronutrient inadequacies despite having sufficient or excessive amounts of calories [52,53,54]. Various chronic diseases, including diabetes, cancer, osteoporosis, and/or cardiovascular diseases, impaired immunity, general fatigue, and cognitive deficits, have been linked to “hidden hunger” [52,55,56]. Overall, poi can provide a rich source of minerals that are easily bioavailable (due to fermentation), without interfering with the absorption of other minerals as compared to mineral supplements. For example, Zn supplements can interfere with Cu, Fe, and Mg absorption and utilization [57,58,59,60,61].

The carbohydrate contents of poi listed on nutritional labels ranged from 14 to 21 g/100 g of poi, which is slightly lower than what is listed on the USDA website at 27 g [48]. Poi is considered a good source of resistant starch (starches resistant to digestion and fermented by the gut microbiome), which can provide potential health benefits, including improved gut health and blood sugar regulation, lowering cholesterol, reducing gallstone formation, increasing mineral absorption, as well as colon cancer prevention via an improvement in insulin sensitivity and the provision of energy for colonic epithelial cells due to their prebiotic effect [20,62,63,64]. RS resists fermentation in the intestine and directly passes to the intestinal colon and is fermented by the colonic microbiota [65].

Studies have shown that the types of fermentation processes can increase RS in foods such as bread [66]. Fermentation of uncooked taro slices by lactic acid bacteria, followed by one cycle of autoclaving and cooling, significantly increased the RS contents of the taro flour [67]. Other processes, such as the cooking–cooling process or steaming, have also increased the RS contents of taro, possibly due to alterations in the microstructure of the starch granules [67,68,69].

To our knowledge, this is also the first study to report on the RS and NRS contents of commercial poi brands. In our study, although the RS contents of all five brands were comparable in fresh poi (0 h), fermentation significantly increased RS in four poi brands. Similarly, in our study, NRS was also increased after fermentation in two poi brands. Gram-positive bacteria such as lactic acid bacteria (LAB), present in soil, taro, or the poi production facility, can facilitate the natural fermentation of poi at room temperature [15]. LAB have the ability to ferment sucrose, glucose, and lactose present in foods into lactic acid and are positively correlated with several health benefits, including reducing diabetes burden, improving gut health and microbial dysbiosis, reducing cancer risk, and/or enhancing defenses against infectious diseases through immune modulation [70,71,72,73]. At the order level, *Lactobacillales* was the dominant microbe in all poi brands before and after fermentation, except for Hanalei brand poi, where *Bacillales* represented the majority of the microbial diversity before and after fermentation. Similar shifts in bacterial communities have also been noted in taro fermented for 24 h, including shifts in *Bacteriodes* and *Firmicutes* phyla [22].

At the genus level, *Leuconostoc*, *Bacillus*, *Lactococcus*, *Weissella,* and *Lactobacillus* dominated the bacterial communities in both fresh and fermented poi. These LAB also form the core microbiota of other fermented foods, such as kimchi and sourdough bread [74]. Our findings are also supported by other studies that identified *Lactobacillus* and *Streptococcus* (now *Lactococcus*) from taro cooked without the peels [75] and were found to predominate the fermented commercial poi analyzed from Honolulu in 1994 [27].

*Lactobacillus* has demonstrated antioxidant properties, while *Weissella* has exhibited anti-obesity and anti-inflammatory properties [76,77]. *Lactobacillales* and *Bacillales* microbes belong to the phyla *Firmicutes*, which, along with the phylum Bacteroidetes, represent approx. 90% of the human gut microbiota [78]. A cross-over clinical study investigating the probiotic effects of poi consumption among 18 healthy individuals determined that consuming 130 gm of fresh poi three times a day for four weeks had no significant differences in the gut microbiome as compared to a control diet [14]. Further studies are warranted to identify the probiotic effects of fermented poi and for longer consumption periods. Direct health benefits of poi consumption, such as mineral bioavailability, glucose metabolism (due to high carbohydrate contents), and other health parameters, require further investigation.

Alpha and beta diversity analyses further underscored the variability in the microbial community structure among individual poi brands. Notably, Pomai poi demonstrated significant shifts in both alpha diversity (Chao1, Shannon, and Simpson indices) and beta diversity between fresh and fermented samples, suggesting that fermentation exerts a more pronounced effect on this brand compared to others [43]. The higher F-values observed for Pomai indicate that the microbial restructuring is unlikely to be random but rather reflects selective microbial succession during fermentation. These findings suggest that fermentation dynamics are brand-specific and highlight the importance of considering the starting microbial composition when evaluating the ecological and nutritional properties of fermented poi [20].

Univariate and multivariate analyses of global metabolite signatures revealed distinct biochemical profiles both across poi brands and between fresh and fermented states. In fresh poi, the presence of multiple free sugars, organic acids, fatty acids, tocopherols, flavonoids, and 18 amino acids underscores its rich nutrient composition [37]. However, fermentation led to significant brand-specific changes, with hierarchical clustering analyses and heatmaps showing clear separations, particularly for Aloha poi compared to the Hanalei, Kokua, Pomai, and Taro brands. These data indicate that while poi consistently provides diverse metabolites, fermentation modulates these metabolite patterns in ways that may influence both its nutritional value and sensory qualities [40].

The PLS-DA score plots further supported these findings by demonstrating clear clustering of metabolite profiles according to brand and fermentation stage [41]. The distinct separation of samples over time suggests that fermentation drives reproducible shifts in metabolic signatures, regardless of baseline variation among brands. Reductions in free sugars together with increases in organic acids such as citric, lactic, and maleic acid reflect the metabolic activity of lactic acid bacteria and other fermenting microbes. These shifts not only contribute to the characteristic sour flavor of fermented poi but may also enhance its functional properties, given the potential roles of organic acids in gut microbiota modulation and metabolic health [79].

Metabolomic studies revealed the presence of both essential and non-essential amino acids in all poi, along with essential fatty acids, vitamin E, and flavanols. These nutrients are critical for various physiological functions, including muscle repair, immune support, and antioxidative defense. While our findings suggest that poi retains these nutrients even after fermentation, it is important to note that fermentation may also influence their concentration and bioavailability. For example, microbial activity could degrade or transform some amino acids and vitamins, potentially impacting their nutritional efficacy. Furthermore, the sour taste associated with fermented poi may represent a barrier to widespread acceptance, as it is often considered an acquired taste. This underscores the importance of cultural context in the perception and consumption of traditionally fermented foods. Interestingly, we did not find any strong correlations among fermenting bacteria and metabolites.

Fermentation also altered the abundance of amino acids, with increases particularly evident in Aloha and Kokua poi. Essential amino acids such as glutamine, histidine, lysine, isoleucine, leucine, and valine were significantly elevated following fermentation, suggesting that microbial proteolysis and metabolic activity contribute to their enrichment [24]. These changes are nutritionally meaningful, as they may increase the bioavailability of key amino acids despite poi’s relatively low total protein content [25]. At the same time, decreases in amino acids such as aspartic acid, proline, serine, and methionine in some brands suggest microbial utilization of certain substrates during fermentation. Together, these results indicate a complex interplay between microbial metabolism and amino acid availability in poi.

Pearson correlation analyses between microbial taxa and global metabolites provided further insight into the microbial–metabolite relationships during fermentation [42]. Although no single bacterial group was consistently associated with metabolite changes across all brands, significant correlations were identified between several bacterial orders and amino acids, sugars, and organic acids. These associations highlight the context-dependent nature of microbial metabolism in poi fermentation and may explain the brand-specific differences observed in both microbial succession and metabolite profiles. Such findings emphasize the importance of integrating microbial ecology with metabolomic data to better understand the nutritional and functional outcomes of traditional fermented foods like poi [44].

Overall, all commercial poi brands evaluated in our study provided a good source of several minerals, RS, free sugars, fatty acids, flavonoids (epicatechin and catechin), vitamin E (α and γ tocopherols), and a total of 18 amino acids that included nine essential and nine non-essential amino acids. Fermentation at 24 and 48 h improved the nutritional value of most poi by increasing RS (in all poi except Aloha), increasing NRS in Hanalei and Kokua poi, increasing beneficial microbes (in all poi), and increasing essential and non-essential amino acids in Aloha and Kokua poi. Fermented poi can be a good choice as one of the first foods introduced to babies, as it provides several amino acids, including nine essential amino acids, which contain several minerals and beneficial LAB that can help populate the infant gut microbiome and allow easy absorption of minerals and phytonutrients. Fermented poi can also provide several health-beneficial nutrients at all stages of development, including adulthood, as well as help mitigate health conditions such as celiac disease among adults.

A limitation of this study is that our nutrient measurements were reported in relative concentrations. To provide a more accurate understanding of the nutritional profile and how fermentation affects it, future studies should focus on quantifying the absolute values of amino acids, vitamins, and other bioactive compounds. This would enable a clearer comparison between unfermented and fermented poi and facilitate nutritional labeling for consumer products. While there is growing interest in functional fermented foods, a potential concern is the commodification of traditional foods like poi, which may lead to their transformation into niche or elite products. This raises questions about food equity and access, particularly for indigenous communities for whom poi holds cultural and dietary significance. Promoting naturally fermented poi supports food sovereignty and aligns with sustainable and traditional food systems.

## 5. Conclusions

Our study provides a foundational understanding of natural fermentation on the nutritional and biochemical properties of commercial poi brands from Hawai‘i. Fermentation of poi at 25 °C for 24 h to 48 h significantly increased RS, NRS, and some of the amino acids, including essential amino acid contents of poi. However, fermentation had no effect on essential fatty acids, vitamin E, and flavanols or the mineral contents of poi. It is possible that the microbial shifts (increase in beneficial LAB) in fermented poi may contribute to increased bioavailability of minerals and flavanols. In our study, fermentation times of 24 h and 48 h improved the nutritional value of most poi brands. However, it is difficult to predict an optimal fermentation time due to differences in personal taste and palatability of fermented poi. Future studies using controlled fermentation conditions with specific probiotic bacterial strains and palatability tasting are warranted, along with the elucidation of in vivo bioavailability of minerals from fermented poi. Such studies could delineate how targeted microbial communities influence the metabolic landscape of the food, ultimately contributing to its health benefits or sensory attributes. Ongoing and future investigations will explore other traditional and novel fermented foods to assess their health-promoting benefits. Such studies are expected to preserve traditional food practices in contemporary health paradigms.

## Figures and Tables

**Table 1:** Nutritional composition and moisture contents of commercial poi brands.

Nutrition Facts *	Aloha Brand	Hanalei Brand	Kokua Brand	Pomai Brand	Taro Brand
Serving size (g)	90	104	NA	NA	82
Total fat (g)	0	0.5	NA	NA	0
Saturated fat (g)	0	0	NA	NA	0
Total carbohydrate (g)	19	14	NA	NA	12
Dietary fiber (g)	2	1	NA	NA	1
Sugars (g)	0	1	NA	NA	0
Protein (g)	1	0	NA	NA	0
Vitamin A	0%	NA	NA	NA	NA
Vitamin C	0%	NA	NA	NA	NA
Vitamin D	NA	0 mcg	NA	NA	0 mcg
Calcium	2% DV	20 mg	NA	NA	16 mg/2% DV
Iron	4% DV	0.8 mg DV	NA	NA	1 mg
Potassium	NA	120 mg DV	NA	NA	107 mg DV
Laboratory-measured moisture content (%) **	80.74 ± 1.131 ^a^	83.89 ± 0.683 ^a,c^	76.37 ± 0.191 ^b^	84.44 ± 2.060 ^c^	81.20 ± 1.506 ^a,c^

* Each commercial poi brand displayed its own nutrition label. The statistical significance for each nutritional value could not be analyzed since the nutritional labels were the same regardless of purchase dates. ** the moisture content was not provided on nutritional labels but depicts the values analyzed at the University of Hawai‘i Agriculture Diagnostic Service Center (ADSC) in triplicate. NA = not available; DV = daily value. ^a,b,c^ mean values with common letters do not differ (*p* < 0.05).

**Table 2:** Mineral contents of poi.

Minerals	Minerals—Average RDA, AI, and UL (mg/day) in Males and Females (19–70 y)	Minerals in Aloha Poi mg/100 g (*n* = 3)			Minerals in Hanalei Poi mg/100 g (*n* = 3)			Minerals in Kokua Poi mg/100 g (*n* = 3)			Minerals in Pomai Poi mg/100 g (*n* = 3)			Minerals in Taro Poi mg/100 g (*n* = 3)		
		0 h	24 h	48 h	0 h	24 h	48 h	0 h	24 h	48 h	0 h	24 h	48 h	0 h	24 h	48 h
P ^1^	700 * 1250 **	255.1 ± 45.33 ^a,b,d^	255.7 ± 26.23 ^a,b,d^	259 ± 36.77 ^a,b,d^	207.1 ± 3.678 ^a^	217.4 ± 15.68 ^a^	225.1 ± 15.19 ^a,c^	294.6 ± 10.99 ^b^	288.5 ± 3.433 ^b,c^	291.1 ± 9.157 ^b,c^	205.6 ± 10.47 ^d^	201.5 ± 14.5 ^d^	196.1 ± 10.3 ^d^	258.4 ± 33.84 ^a,d^	242.2 ± 25.9 ^a,d^	248.4 ± 29.24 ^a,d^
K ^2^	2600 * 4700 **	998.5 ± 196.7 ^a^	974.9 ± 129.7 ^a^	1014 ± 199 ^a^	1321 ± 80.64 ^a,b^	1380 ± 51.28 ^a,b^	1403 ± 91.61 ^a,b^	1691 ± 28.08 ^b^	1667 ± 16.6 ^b^	1682 ± 34.82 ^b^	1320 ± 285.4 ^a,b^	1309 ± 375.6 ^a,b^	1258 ± 309.7 ^a,b^	1629 ± 241.2 ^b^	1521 ± 166.2 ^a,b^	1541 ± 130.9 ^a,b^
Ca ^1^	1000 * 1300 **	204.8 ± 36.52 ^a^	204.1 ± 19.24 ^a^	205.7 ± 26.29 ^a^	162.1 ± 22.87 ^a^	168.3 ± 18 ^a^	168.3 ± 21.54 ^a^	181.2 ± 19.65 ^a^	177.9 ± 7.16 ^a^	175.4 ± 8.571 ^a^	163.9 ± 29.42 ^a^	168.6 ± 28.51 ^a^	166.4 ± 26.18 ^a^	185.1 ± 41.14 ^a^	175.6 ± 38.44 ^a^	179.6 ± 43.05 ^a^
Mg ^1^	310–320 * (female) 400–420 * (males) 420 **	228.7 ± 30.24 ^a^	229.5 ± 12.09 ^a^	233.2 ± 17.92 ^a^	148.9 ± 14.81 ^b^	157.6 ± 12.49 ^b^	161.2 ± 10.88 ^b^	169.3 ± 6.574 ^b^	166.5 ± 4.775 ^b^	166.4 ± 6.574 ^b^	167.3 ± 11.82 ^b^	169.3 ± 11.42 ^b^	161.6 ± 6.499 ^b^	201.6 ± 22.36 ^a^	189.6 ± 17.62 ^a,b^	193.3 ± 19.57 ^a,b^
Na ^4^	2300 * 2300 **	74.52 ± 7.582 ^a,b,c^	89.7 ± 38.09 ^a,c^	73.42 ± 10.51 ^a,b,c^	53.55 ± 11.18 ^a,b^	57.8 ± 9.769 ^a,b,c^	57.86 ± 10.12 ^a,b,c^	104.5 ± 24.06 ^c^	97.35 ± 15.16 ^a,c^	97.97 ± 15.39 ^a,c^	32 ± 11.54 ^b^	34.9 ± 16.87 ^b^	30.51 ± 11.71 ^b^	57.24 ± 7.063 ^a,b^	52.38 ± 3.741 ^a,b^	51.51 ± 6.335 ^a,b^
Fe ^1^	18 * (female) 8 * (males) 18 **	10.99 ± 4.451 ^a^	10.84 ± 3.405 ^a^	12.03 ± 4.655 ^a^	6.287 ± 0.516 ^a^	6.746 ± 0.792 ^a^	6.773 ± 0.866 ^a^	10.16 ± 2.041 ^a^	10.31 ± 1.458 ^a^	10.74 ± 0.973 ^a^	8.001 ± 1.012 ^a^	7.838 ± 2.328 ^a^	7.811 ± 1.095 ^a^	8.138 ± 0.97 ^a^	7.765 ± 1.079 ^a^	7.962 ± 1.163 ^a^
Mn ^2^	2.3 **	6.923 ± 4.247 ^a^	6.806 ± 3.834 ^a^	6.982 ± 3.919 ^a^	2.736 ± 2.369 ^a^	2.812 ± 2.347 ^a^	2.883 ± 2.379 ^a^	2.466 ± 0.148 ^a^	2.394 ± 0.129 ^a^	2.367 ± 0.117 ^a^	3.107 ± 0.239 ^a^	3.15 ± 0.384 ^a^	3.041 ± 0.427 ^a^	6.238 ± 1.111 ^a^	5.903 ± 1.369 ^a^	6.003 ± 1.154 ^a^
Zn ^1^	8 * (female) 11 * (males) 11 **	8.69 ± 2.869 ^a,c^	7.717 ± 1.972 ^a,c^	7.036 ± 1.786 ^a,c^	3.148 ± 1.153 ^b^	4.304 ± 0.881 ^a,b^	4.093 ± 1.683 ^a,b^	10.46 ± 2.453 ^c^	10.06 ± 2.349 ^c,d^	10.94 ± 3.754 ^c^	4.88 ± 0.686 ^a,d^	5.593 ± 1.101 ^a,c,d^	5.141 ± 1.104 ^a,c,e^	5.547 ± 0.299 ^a,c,d^	4.785 ± 0.744 ^a,d^	4.777 ± 0.584 ^a,d^
Cu ^1^	0.9 **	5.747 ± 5.663 ^a^	2.89 ± 0.441 ^a^	2.741 ± 0.549 ^a^	3.287 ± 1.092 ^a^	3.495 ± 0.635 ^a^	3.4 ± 0.547 ^a^	2.528 ± 0.777 ^a^	2.419 ± 0.994 ^a^	2.647 ± 1.711 ^a^	3.65 ± 0.324 ^a^	3.518 ± 0.662 ^a^	3.449 ± 0.305 ^a^	3.925 ± 0.479 ^a^	4.086 ± 0.589 ^a^	3.75 ± 0.468 ^a^
B ^3^	1.5 ***	0.677 ± 0.153 ^a,b^	0.603 ± 0.209 ^a,b^	0.655 ± 0.099 ^a,b^	0.401 ± 0.017 ^a^	0.447 ± 0.059 ^a^	0.453 ± 0.039 ^a^	0.746 ± 0.04 ^b,c^	0.751 ± 0.1 ^b,c^	0.825 ± 0.158 ^b^	0.517 ± 0.013 ^a,c^	0.508 ± 0.037 ^a,c^	0.49 ± 0.076 ^a,c^	0.625 ± 0.139 ^a,b^	0.524 ± 0.072 ^a,b^	0.509 ± 0.078 ^a^
Mo		0.19 ± 0.24 ^a^	0.06 ± 0.10 ^a^	0.07 ± 0.08 ^a^	0.03 ± 0.03 ^a^	0.04 ± 0.03 ^a^	0.05 ± 0.05 ^a^	0.18 ± 0.09 ^a^	0.13 ± 0.06 ^a^	0.16 ± 0.15 ^a^	0.07 ± 0.03 ^a^	0.05 ± 0.03 ^a^	0.03 ± 0.04 ^a^	0.21 ± 0.30 ^a^	0.05 ± 0.04 ^a^	0.05 ± 0.04 ^a^

^1^ Recommended Dietary Allowance (RDA); ^2^ Adequate Intake (AI); ^3^ Tolerable Upper Intake Level (UL); ^4^ CDRR = Chronic Disease Risk Reduction Level; * sourced from the Dietary Guidelines for Americans, 2020–2025; ** FDA.gov (accessed on 24 November 2024); *** NIH.gov: The Food and Nutrition Board has not established an RDA or AI for boron [2], and boron does not have a DV. Total median boron intakes from dietary supplements and foods are about 1.0 to 1.5 mg/day for adults., ^a^,^b^,^c^,^d^ mean values with common letters do not differ (*p* < 0.05).

**Table 3:** Alpha and beta diversity for individual poi brands (order level).

Poi Type	Chao1	Shannon	Simpson	Beta Diversity
	*p*-Value (F-Value)			*p*-Value (F-Value); R-Squared
Aloha Poi	0.1517 (2.625)	0.054635 (4.9061)	0.72472 (0.33988)	0.135 (2.3467); 0.43891
Hanalei Poi	0.00036443 (39)	0.27816 (1.60)	0.5652 (0.62845)	0.143 (1.7287); 0.36557
Kokua Poi	0.49205 (0.8)	0.94387 (0.058322)	0.93635 (0.066494)	0.718 (0.1632); 0.051592
Pomai Poi	0.031491 (6.5)	0.0032237 (17.308)	0.0018695 (21.353)	0.035 (15.118); 0.83441
Taro Poi	0.46704 (0.86667)	0.37857 (1.147)	0.47933 (0.83334)	0.062 (2.3996); 0.44441

**Table 4:** Univariate analysis at the order level.

Poi Type	Bacteria	log2FC	logCPM	*p*-Values	FDR
Aloha (24 h vs. 0 h)	*Acetobacterales*	2.6945	18.791	0.0019061	0.026685
Aloha (48 h vs. 0 h)	*Acetobacterales*	2.6894	18.791	0.0019396	0.027155
Hanalei (24 h vs. 0 h)	*Bacillales*	5.0053	20.965	4.6694 × 10^−5^	3.7355 × 10^−4^
	*Lactobacillales*	9.947	19.499	1.0571 × 10^−4^	4.2284 × 10^−4^
	*Paenibacillales*	9.2845	15.897	1.8001 × 10^−4^	4.8003 × 10^−4^
	*Rickettsiales*	−3.8424	9.5277	0.012311	0.024622
Hanalei (48 h vs. 0 h)	*Bacillales*	5.8261	20.965	5.0368 × 10^−6^	4.0295 × 10^−5^
	*Lactobacillales*	9.2047	19.499	2.2913 × 10^−4^	9.1651 × 10^−4^
	*Paenibacillales*	5.1236	15.897	0.014407	0.023961
	*Rickettsiales*	−6.1149	9.5277	4.668 × 10^−4^	0.0012448
	*Chloroplast*	−4.2247	12.479	0.014975	0.023961
Kokua (24 h vs. 0 h)	*Staphylococcales*	4.1499	8.6133	4.7759 × 10^−4^	0.0057311
Pomai (24 h vs. 0 h)	*Lactobacillales*	5.4469	21.905	0.0023267	0.016287
	*Pseudomonadales*	−5.2523	13.232	0.013722	0.048025
Pomai (48 h vs. 0 h)	*Pseudomonadales*	−5.4531	13.232	0.011211	0.04091
	*Acetobacterales*	−6.6853	8.9206	0.011689	0.04091
	*Lactobacillales*	3.9097	21.905	0.019219	0.044845
Taro (24 h vs. 0 h)	*Chloroplast*	−7.1425	18.653	1.5086 × 10^−4^	0.0019612
	*Rickettsiales*	−6.5874	14.251	7.3087 × 10^−4^	0.0047507
	*Veillonellales_Selenomon*	−7.8672	8.2966	0.0038517	0.016691
Taro (48 h vs. 0 h)	*Chloroplast*	−7.0509	18.653	1.7311 × 10^−4^	0.0022504
	*Bacillales*	−7.1661	9.8968	0.016601	0.043163
	*Rickettsiales*	−6.5583	14.251	7.6878 × 10^−4^	0.0049971
	*Veillonellales_Selenomon*	−6.5424	8.2966	0.012738	0.0414
	*Acetobacterales*	6.7157	15.951	0.0064293	0.02786
	*Lactobacillales*	3.2676	19.818	0.022279	0.048271

**Table 5:** Univariate analysis at the genus level.

Poi Type	Bacteria	log2FC	logCPM	*p*-Values	FDR
Aloha (48 h vs. 0 h)	*Erwinia*	−7.3815	9.6925	3.7464 × 10^−4^	0.014986
	*Lacticaseibacillus*	4.8789	9.9958	7.5536 × 10^−4^	0.015107
	*Liquorilactobacillus*	3.8105	16.004	0.0029049	0.038733
	*Ameyamaea*	4.7979	11.043	0.0054701	0.045588
	*Gluconobacter*	3.5008	14.47	0.0056986	0.045588
Hanalei (24 h vs. 0 h)	Not_Assigned	−4.6964	13.539	0.0017626	0.0098498
	*Streptococcus*	11.659	19.252	0.001947	0.0098498
	*Paenibacillus*	7.9309	15.999	0.0026863	0.0098498
	*Bacillus*	5.5402	20.343	0.023304	0.042725
	*Leuconostoc*	3.4136	12.256	0.016102	0.042725
	*Geobacillus*	−3.2936	14.119	0.022835	0.042725
Hanalei (48 h vs. 0 h)	Not_Assigned	−6.4615	13.539	7.0012 × 10^−5^	7.7013 × 10^−4^
	*Streptococcus*	10.221	19.252	0.004495	0.018986
	*Bacillus*	5.5857	20.343	0.022453	0.049396
	*Leuconostoc*	3.4245	12.256	0.015767	0.043359
	*Geobacillus*	−4.2101	14.119	0.005178	0.018986

## Data Availability

All related data is provided in the manuscript or supporting materials.

## Supplementary Materials

The following supporting information can be downloaded at https://www.mdpi.com/article/10.3390/metabo15110748/s1.

## Abbreviations

The following abbreviations are used in this manuscript:

ACN	Acetonitrile
ADSC	Agriculture Diagnostic Service Center
AI	Adequate Intake
ALEX-CIS GCTOF	Automated liner exchange cold injection system gas chromatography time of flight
AMG	Amyloglucosidase
ANOVA	Analysis of variance
ASGPB	Advanced Studies in Genomics, Proteomics and Bioinformatics
ASV	Amplicon sequence variants
bp	Base pair
CDRR	Chronic Disease Risk Reduction Level
DADA2	Deficiency of Adenosine Deaminase 2
DNA	Deoxyribonucleic acid
dsDNA	Double-stranded Deoxyribonucleic acid
DV	Daily value
EI	Electron ionization
FAME	Fatty acid methyl ester
FDR	False discovery rate
GC	Gas chromatography
GOPOD	Glucose Determination Reagent
HCA	Hierarchical cluster analysis
IPA	Isopropanol
LAB	Lactic acid bacteria
LSD	Least Significant Difference
MaxEE	Maximum expected error
MS	Mass spectrometer
MSTFA	*N*-methyl-*N*-(trimethylsilyl) trifluoroacetamide
NA	Not available
NIH	National Institutes of Health
NRS	Non-resistant starch
OPLS-DA	Orthogonal partial least squares discriminant analysis
OTU	Operational Taxonomic Unit
PCA	Principal component analysis
PCR	Polymerase chain reaction
PERMANOVA	Permutational analysis of variance
PLS-DA	Partial least squares discriminant analysis
RCF	Relative centrifugal force
RDA	Recommended Dietary Allowance
RDP	Ribosomal Database Project
rRNA	Ribosomal ribonucleic acid
RS	Resistant starch
TMM	Trimmed mean of M
TOF	Time-of-flight
UHM	UH Mānoa
UL	Tolerable Upper Intake Level
